# Striatal dopamine tone is positively associated with adiposity in humans as determined by PET using dual dopamine type-2 receptor antagonist tracers

**DOI:** 10.1038/s41380-025-02960-y

**Published:** 2025-04-06

**Authors:** Valerie L. Darcey, Juen Guo, Meible Chi, Stephanie T. Chung, Amber B. Courville, Isabelle Gallagher, Peter Herscovitch, Rebecca Howard, Melissa La Noire, Lauren Milley, Alex Schick, Michael Stagliano, Sara Turner, Nicholas Urbanski, Shanna Yang, Eunha Yim, Nan Zhai, Megan S. Zhou, Kevin D. Hall

**Affiliations:** 1 Integrative Physiology Section, National Institute of Diabetes & Digestive & Kidney Diseases, National Institutes of Health, Bethesda, MD USA; 2 Center on Compulsive Behaviors, Intramural Research Program, NIH, Bethesda, MD USA; 3 Diabetes, Endocrinology, and Obesity Branch, National Institute of Diabetes, Digestive, and Kidney Diseases, National Institutes of Health, Bethesda, MD USA; 4 Human Energy and Body Weight Regulation Core, National Institute of Diabetes & Digestive & Kidney Diseases, National Institutes of Health, Bethesda, MD USA; 5 Positron Emission Tomography Department, Clinical Center, National Institutes of Health, Bethesda, MD USA; 6 Nutrition Department, Clinical Center, National Institutes of Health, Bethesda, MD USA; 7 University of Maryland, College Park, MD USA

**Keywords:** Neuroscience, Physiology

## Abstract

The relationship between adiposity and dopamine type-2 receptor binding potential (D2BP) in the human brain has been repeatedly studied for >20 years with highly discrepant results, likely due to variable methodologies and differing study populations. We conducted a controlled inpatient feeding study to measure D2BP in the striatum using positron emission tomography with both [^18^F]fallypride and [^11^C]raclopride in pseudo-random order in 54 young adults with a wide range of body mass index (BMI 20–44 kg/m^2^). Within-subject D2BP measurements using the two tracers were moderately correlated (r = 0.47, *p* < 0.001). D2BP was negatively correlated with BMI as measured by [^11^C]raclopride (r = −0.51; *p* < 0.0001) but not [^18^F]fallypride (r = −0.01; *p* = 0.92) and these correlation coefficients were significantly different from each other (*p* < 0.001). Given that [^18^F]fallypride has greater binding affinity to dopamine type-2 receptors than [^11^C]raclopride, which is more easily displaced by endogenous dopamine, our results suggest that adiposity is positively associated with increased striatal dopamine tone.ClinicalTrials.gov Identifier: NCT03648892

## Introduction

Over 20 years ago, Wang et al. published a seminal paper reporting a negative correlation between body mass index (BMI) in people with obesity and brain dopamine type-2 receptor binding potential (D2BP) measured in the striatum using positron emission tomography (PET) with the radiotracer antagonist [^11^C]raclopride [1]. This finding was interpreted as a decrease in dopamine type-2 receptor number with increased adiposity and suggested that people with obesity have a deficiency in dopamine signaling, thereby sharing neurobehavioral characteristics with people suffering from addiction and compulsive behaviors. Similar results of decreased dopamine type-2 receptors were subsequently reported in a seminal rodent study of diet-induced obesity demonstrating addiction-like reward deficits and compulsive eating [2]. To date, these studies have been cited very widely and have cemented the idea that obesity is linked to a decrease in striatal dopamine D2 receptors. However, subsequent studies in humans have yielded conflicting results, with some studies showing positive associations between adiposity and D2 receptors or D2BP [3–6], others showing negative associations [7, 8], and some showing no association at all [9–12] (as reviewed by [13]). Similarly, several rodent studies have yielded discrepant findings, with some demonstrating that in diet induced obesity, some aspects of D2 receptor biology either decrease [14, 15], increase [16, 17], or remain similar to control animals [15, 18].

Considering the inconsistent findings in humans, some researchers have recently concluded that there is no meaningful relationship between brain dopamine and obesity [19, 20]. Alternatively, differences in methodology may explain discrepancies between the studies. For example, in the human studies, PET scans were conducted at different times of the day in participants that were in different physiological states (e.g., fed or fasted), if at all reported. Critically, studies often use different radiotracers with different pharmacokinetics. For example, while two commonly used radiotracers, [^18^F]fallypride and [^11^C]raclopride, are both D2/3 receptor antagonists, [^18^F]fallypride has greater affinity for the D2/3 R [21] compared to [^11^C]raclopride [22]. The resulting differential sensitivity to competition with endogenous dopamine may lead to different interpretations of the nature of the relationship between brain dopamine and obesity. Furthermore, only one previous study [23, 24] controlled food intake during the days prior to the PET scan, which was recently shown to affect striatal D2BP in people with obesity [25]. Additionally, variations in study populations under investigation – specifically, greater age [6, 26], single sex [27, 28], and limited BMI ranges [3, 12] - may have contributed to the differing results.

A novel hypothesis aimed at explaining the seemingly discrepant human results was proposed by Horstmann et al. who suggested that differences in D2BP between BMI categories are better interpreted as differences in tonic dopamine levels that compete for D2 receptor binding with radiotracers used to measure D2BP [29]. They propose that dopamine tone may decrease as people move from low to moderate BMI thereby allowing more tracer to bind with D2 receptors, which is reflected as an increase in D2BP with BMI. In theory, such a model suggests that as people gain weight and move into moderate BMI range, they might experience heightened reward sensitivity due to an amplified phasic dopamine response on a background of low tonic dopamine. In the moderate BMI range, tonic dopamine levels stop decreasing thereby resulting in a flat part of the D2BP vs BMI curve. However, as BMI further increases, dopamine tone is hypothesized to increase and displace the radiotracer from the D2 receptor thereby decreasing D2BP – a state theoretically coupled with a relatively blunted magnitude of phasic dopamine response and decrease in reward sensitivity. Thus, Horstmann et al. hypothesized that the relationship between D2BP and BMI is a curve with a negative quadratic coefficient. Past studies could potentially be reconciled by noting that each study included subjects from a relatively narrow BMI range and therefore sampled from only a small part of the nonlinear curve relating D2BP with BMI and resulting in conflicting linear relationships [29].

To test the neurochemical component of this theory and avoid potentially confounding factors in previous studies, the main objectives of our preregistered study was to measure striatal D2BP using two common radiotracers under highly standardized conditions in adults aged 18–45 years encompassing a wide range of BMIs. Specifically, the participants were admitted as inpatients to the NIH Clinical Center and we measured striatal D2BP using both [^18^F]fallypride and [^11^C]raclopride in pseudorandom order on separate days in the overnight fasted state following a period of controlled dietary stabilization. The primary aims of the study were to detect both (1) the within subject correlation between whole striatal D2BP assessed by [^18^F]fallypride and [^11^C]raclopride as well as (2) quadratic and linear relationships between whole striatal D2BP and BMI.

## Results

**Fig. 1:**
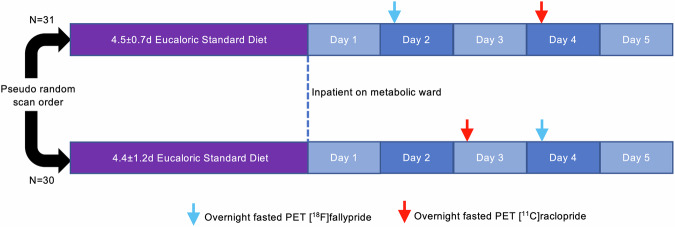
Study design. Sixty-one men and women consumed a provided weight-stabilizing standardized diet for an average of 4.5 ± 1.0 days prior to admission to the NIH Clinical Center for testing. During their inpatient stay, participants continued their dietary stabilization. Between their second day of admission and the morning of their discharge, participants completed morning PET scans in the overnight fasted state with [^18^F]fallypride and [^11^C]raclopride on separate days in pseudo-random order after an average of 6.5 ± 1.3 and 6.8 ± 1.1 total days of dietary stabilization, respectively. Arrows indicate mode value for scan completion day.

Sixty-one weight stable adults across a wide BMI range completed an in-patient admission to the NIH Clinical Center to ensure standardized composition and timing of meals as well as compliance with overnight fasting prior to PET scanning (Table 1; Supplementary Fig. 1). Additional participant information (usual diet, trait eating behavior, stress, depression, life satisfaction and sleep quality) can be found in Supplementary Table 1. Participants were asked to completely consume the provided eucaloric standard diet (50% calories from carbohydrate, 35% from fat, 15% from protein) for 3–5 days in advance of an inpatient admission where they continued the standardized diet for 5 additional days (Fig. 1). During their inpatient admission, participants completed PET neuroimaging in the overnight fasted state with both [^11^C]raclopride and [^18^F]fallypride in pseudorandom order in addition to a high-resolution neuroanatomical magnetic resonance image (MRI).

**Table 1:** Characteristics of participants completing PET scanning with technically adequate images.

	Enrolled participants	[18F] fallypride	[11C] raclopride	[11C] raclopride & [18F] fallypride
N	61	57	56	54
Females	40 (65%)	38 (66.7%)	36 (64.3%)	35 (64.8%)
Age (years)	32.2 ± 7.2	31.8 ± 7.2	31.6 ± 7.1	31.4 ± 7.1
Race				
Black	32 (52.5%)	28 (49.1%)	30 (53.6%)	28 (51.9%)
White	18 (29.5%)	18 (31.6%)	17 (30.4%)	17 (31.5%)
Asian	7 (11.5%)	7 (12.3%)	6 (10.7%)	6 (11.1%)
Other/Multiple	4 (6.6%)	4 (7.1%)	3 (5.4%)	3 (5.6%)
Body weight (kg)				
Mean	85.9 ± 25.3	84.6 ± 24.2	85.0 ± 24.2	84.4 ± 23.6
Range	45.9–148.6	45.9–148.6	45.9–148.6	45.9–148.6
Body fat (%)				
Mean	35.0 ± 12.6	34.7 ± 12.1	34.4 ± 12.0	34.1 ± 12.1
Range	11.3–59.0	11.3–56.7	11.3–52.4	11.3–52.4
BMI (kg/m^2^)				
Mean	30.1 ± 8.2	29.6 ± 7.6	29.7 ± 7.7	29.5 ± 7.5
Range	20.3–52.8	20.3–44.4	20.3–44.8	20.3–44.4

Means and standard deviations indicated.

### Within subject correlation between D2BP measured by [^11^C]raclopride and [^18^F]fallypride

**Fig. 2:**
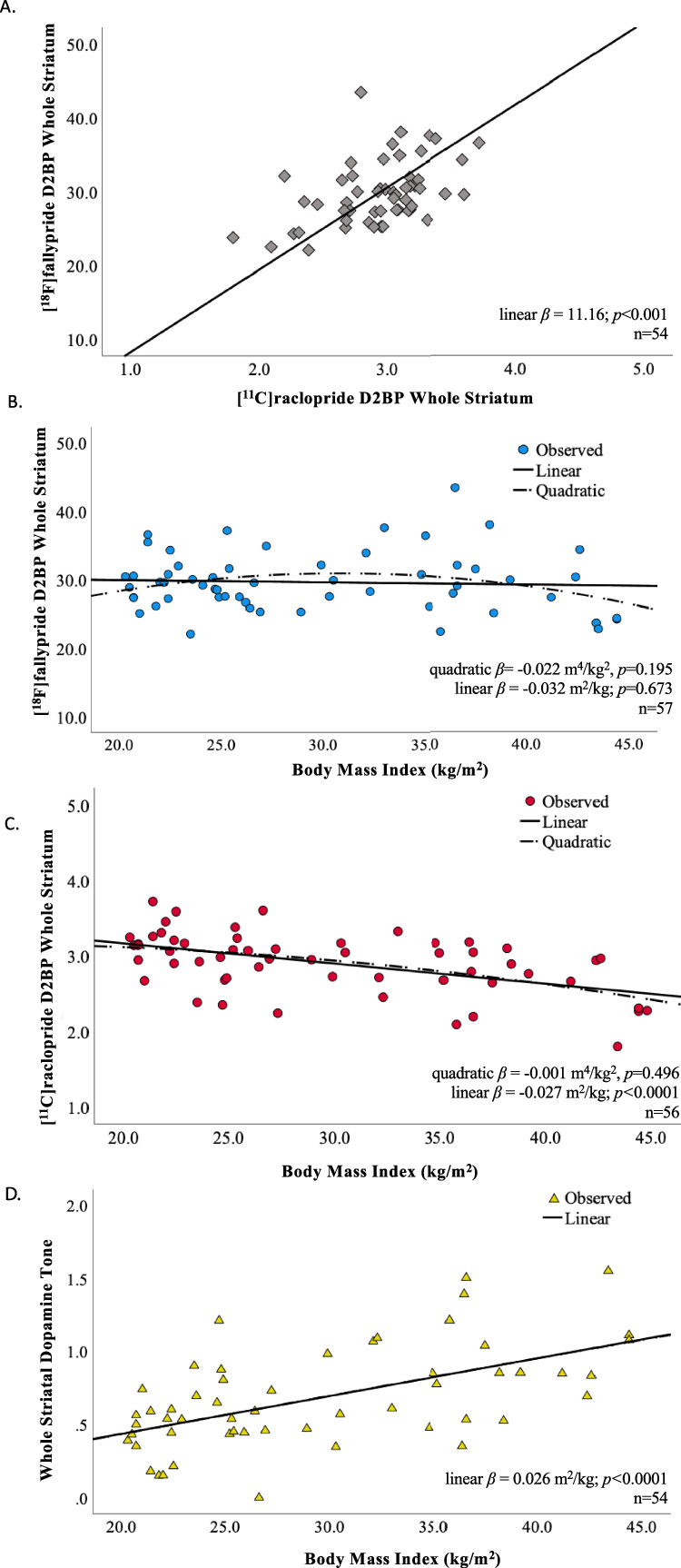
Striatal D2BP as measured within subject by [^18^F]fallypride and [^11^C]raclopride were correlated but differentially related to BMI. **A** Within-subject measurements of D2BP using [^18^F]fallypride and [^11^C]raclopride in the overnight fasted state were correlated using the whole striatum in a region of interest analysis. Trendline and slope parameters reflect standard major axis regression. **B** Whole striatal region of interest analysis using [^18^F]fallypride indicated no significant relationship between D2BP and BMI. **C** Whole striatal region of interest analysis using [^11^C]raclopride indicated a negative linear relationship between BMI and D2BP, but no significant quadratic relationship. **D** Index of dopamine tone across the whole striatum is linearly correlated with BMI (*n* = 54).

Both D2BP_raclo_ and D2BP_fally_ were measured during a period of dietary stabilization and after confirmed overnight fasts of similar duration. Across all participants with both scans meeting quality control requirements (*n* = 54), D2BP_raclo_ and D2BP_fally_ were positively correlated within the region of interest (ROI) defining the whole striatum (r = 0.468, *p* < 0.001) (Primary Aim 1; Fig. 2A). Exploratory ROI analyses within bilateral striatal structures including caudate, putamen, accumbens and pallidum revealed significant within-subject positive correlations between D2BP_raclo_ and D2BP_fally_ in caudate (r = 0.467, *p* < 0.001), putamen (r = 0.547, *p* < 0.001), accumbens (r = 0.484, *p* < 0.001) and pallidum (r = 0.685, *p* < 0.001) (Supplementary Fig. 2). Given the exploratory nature of these and subsequently presented individual ROI analyses, we present all pertinent analyses and no adjustment for multiple comparisons was performed.

Voxelwise analyses within the striatal mask supported the ROI results, revealing clusters where D2BP_raclo_ and D2BP_fally_ were positively correlated in regions spanning dorsal and ventral striatum (Supplementary Fig. 3; Supplementary Table 2).

### BMI was not related to striatal D2BP measured with [^18^F]fallypride but was negatively linearly related to striatal D2BP measured with [^11^C]raclopride

The mean whole striatal D2BP as measured by [^18^F]fallypride was neither quadratically (*p* = 0.195) nor linearly (*p* = 0.673) associated with BMI (Fig. 2B). These results were robust to the exclusion of data from one participant whose striatal D2BP measured by [^18^F]fallypride was determined to be an outlier [30] (Supplementary Fig. 4). In contrast, mean whole striatal D2BP measured using [^11^C]raclopride decreased linearly with increasing BMI (r = −0.514, *p* < 0.0001) but was not quadratically associated with BMI (*p* = 0.496) in the same participants under the same scanning conditions (Fig. 2C). No outliers were detected for striatal D2BP measured using [^11^C]raclopride.

**Fig. 3:**
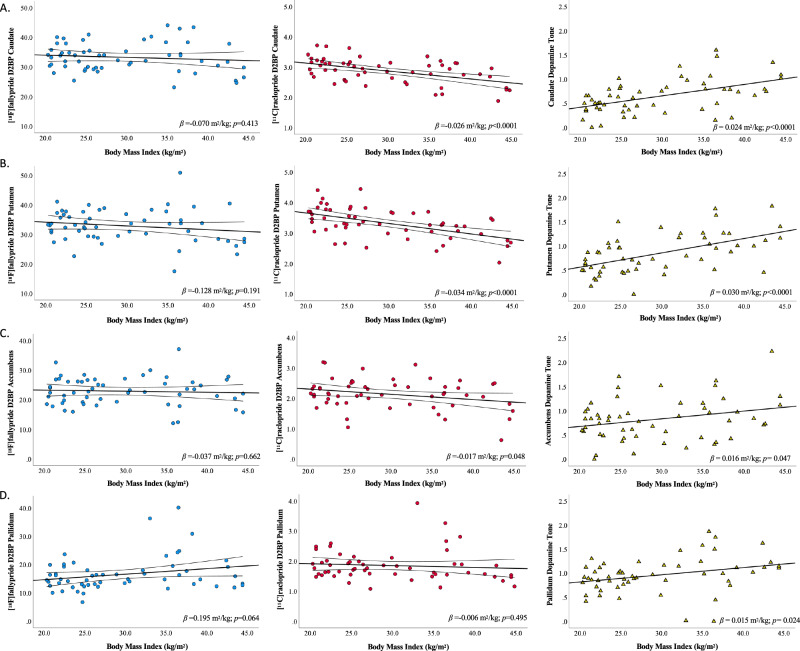
Relationship between BMI and D2BP as measured by [^18^F]fallypride and [^11^C]raclopride, and an index of dopamine tone across striatal regions of interest. Bilateral striatal region of interest analyses reflecting relationships between BMI and D2BP as measured by [^18^F]fallypride (blue circles, left column; *n* = 57) and [^11^C]raclopride (red circles, center column; *n* = 56) in **A** caudate, **B** putamen, **C** accumbens, and **D** pallidum. Regression line and 95%CI indicated. Relationship between BMI and regional indices of dopamine tone (yellow triangles; right column, *n* = 54). ROI analyses are uncorrected for multiple comparisons.

Exploratory analyses within striatal sub-regions of interest support the primary finding that BMI was not significantly related to D2BP as measured by [^18^F]fallypride (Fig. 3, **left column**). Age was negatively correlated with D2BP_fally_ across the whole striatum (r = −0.341, *p* = 0.009), but adjustment for age and sex did not impact the lack of relationship between [^18^F]fallypride D2BP and adiposity using either BMI (Supplementary Fig. 5, **left column**) or percent body fat (Supplementary Figs 6 & 7, **left columns**). Exploratory analyses within striatal sub-regions of interest reveal significant negative linear relationships between BMI and D2BP_raclo_ were observed in all striatal subregions of interest except the pallidum (Fig. 3, **center column**). Age was also negatively correlated with D2BP_raclo_ across the whole striatum (r = −0.452, *p* < 0.001), yet the significance of the relationships between BMI and D2BP_raclo_ across the striatum as a whole and dorsal striatum (caudate and putamen) persisted after adjustment for age and sex (Supplementary Fig. 5, **center column**).

Percent body fat was not significantly linearly related with D2BP_fally_ (Supplementary Fig. 6, **left column**) but was negatively related to D2BP_raclo_ within the whole striatum (r = −0.337, *p* = 0.011) and putamen (r = −0.367, *p* = 0.005) (Supplementary Fig. 6, **center column**). While beta coefficients remain negative after adjustment for age and sex, the *p* values were greater than 0.05 (Supplementary Fig. 7, **center column**). However, both D2BP_fally_ and D2BP_raclo_ were significantly quadratically related to percent body fat (Supplementary Fig. 6, **left and center columns)** regardless of adjustment for age and sex (Supplementary Fig. 7, **left and center columns**). Due to the exploratory nature of these analyses, we did not adjust for multiple comparisons.

Exploratory voxelwise analyses of D2BP were restricted to within the striatal region mask (small volume correction) and revealed that D2BP as measured by [^18^F]fallypride was positively related to BMI in two clusters (proximal to right and left caudate) and negatively related to BMI in the right thalamus. However, only the cluster proximal to the right caudate marginally survived correction for multiple comparisons using AFNI’s 3DClustSim (Supplementary Fig. 8; Supplementary Table 2).

Exploratory voxelwise analyses of D2BP within the striatal mask using [^11^C]raclopride supported the ROI analyses and revealed clusters with peaks where D2BP negatively correlated with BMI in the left and right putamen surviving correction for multiple comparisons (Supplementary Fig. 9; Supplementary Table 2).

### The linear relationship between D2BP_raclo_ and BMI is significantly more negative than between D2BP_fally_ and BMI

A statistical comparison of standardized correlation coefficients of D2BP_raclo_ with BMI and D2BP_fally_ with BMI from 54 participants with both scans showed that the linear relationship between D2BP_raclo_ and BMI was significantly more negative than that of the D2BP_fally_ relationship with BMI (Table 2). Voxelwise maps reflecting the difference in correlation coefficients between the *D2BP*_*raclo*_*× BMI* correlation and *D2BP*_*fally*_*× BMI* correlation produced a cluster in the left caudate-putamen boundary which survived multiple comparisons correction, suggesting this region has the greatest difference in correlation coefficients (Supplementary Fig. 11; Supplementary Table 2).

**Table 2:** Standardized correlation coefficients (β) between measures of adiposity and D2BP or an index of dopamine tone across regions of interest for *n* = 54 participants with both radiotracer scans available.

	*D2BP*_*fally*_	*p*	*D2BP*_*raclo*_	*p*	*p (D2BP*_*fally vs*._ *D2BP*_*raclo*_*)*	*DA Tone*	*p*
*BMI*							
Whole striatum	−0.013	0.923	−0.509	<0.001	<0.001	0.569	<0.001
Caudate	−0.066	0.635	−0.482	<0.001	0.002	0.510	<0.001
Putamen	−0.132	0.343	−0.536	<0.001	0.001	0.554	<0.001
Accumbens	0.000	0.999	−0.238	0.083	0.091	0.272	0.047
Pallidum	0.245	0.074	−0.056	0.687	0.007	0.308	0.024
*Percent Body Fat*							
Whole striatum	−0.056	0.688	−0.314	0.021	0.068	0.326	0.016
Caudate	−0.026	0.851	−0.192	0.164	0.247	0.204	0.140
Putamen	−0.131	0.345	−0.350	0.010	0.089	0.332	0.014
Accumbens	−0.072	0.602	−0.182	0.188	0.437	0.168	0.226
Pallidum	0.039	0.778	−0.131	0.345	0.125	0.217	0.115
*Adjusted for age and sex*							
*BMI*							
Whole striatum	0.141	0.309	−0.337	0.013	0.001	0.441	<0.001
Caudate	0.093	0.503	−0.326	0.016	0.004	0.406	0.002
Putamen	0.002	0.987	−0.382	0.004	0.003	0.443	<0.001
Accumbens	0.095	0.497	−0.162	0.241	0.071	0.234	0.089
Pallidum	0.259	0.059	0.055	0.692	0.063	0.173	0.212
*Percent Body Fat*							
Whole striatum	0.104	0.453	−0.188	0.174	0.043	0.260	0.058
Caudate	0.119	0.393	−0.155	0.262	0.058	0.235	0.087
Putamen	0.022	0.873	−0.222	0.106	0.065	0.271	0.047
Accumbens	0.075	0.590	–0.047	0.735	0.391	0.094	0.501
Pallidum	0.139	0.316	0.025	0.859	0.302	0.099	0.474

D2BP and adiposity measures normalized to facilitate comparison between tracers (Corresponding figures located in Supplementary Figs 8–11).

### An exploratory index of dopamine tone increases linearly with adiposity

Because [^18^F]fallypride binds with higher affinity to D2 type receptors as compared to [^11^C]raclopride [21, 22], the D2BP measured using [^18^F]fallypride likely provides a better index of D2 receptor density while D2BP measured using [^11^C]raclopride is influenced to a greater extent by endogenous dopamine. As detailed in the Methods, we use these assumptions to propose an index of dopamine tone at the individual level by adjusting the [^11^C]raclopride D2BP for the [^18^F]fallypride D2BP to approximately account for the influence of D2 receptor density on the [^11^C]raclopride signal. Specifically, individuals with high dopamine tone were identified as having low [^11^C]raclopride D2BP after adjusting for receptor density (approximated by [^18^F]fallypride D2BP). Thus, a positive index of regional dopamine tone for each participant was calculated by subtracting the adjusted [^11^C]raclopride D2BP value from the group maximum adjusted [^11^C]raclopride D2BP.

Using the Whole Striatal ROI, dopamine tone increased linearly with BMI (r = 0.569, *p* < 0.0001, *n* = 54) (Fig. 2D). This relationship is evident across each sub-region of interest, though predominantly in dorsal striatum (Fig. 3, **right column;** Table 2), where it also persists after adjustment for age and sex (Supplementary Fig. 5, **right column**; Table 2). While the index of whole striatal dopamine tone also increases with percent body fat (r = 0.326, *p* = 0.016; Supplementary Fig. 6; Table 2), only the index of dopamine tone in putamen remains significant after adjustment for age and sex (Supplementary Fig. 7; Table 2).

Voxelwise analyses of calculated dopamine tone within the striatal mask supported the main results and revealed clusters with peaks where dopamine tone is positively correlated with BMI in the bilateral putamen surviving corrections for multiple comparisons (Supplementary Fig. 10; Supplementary Table 2).

## Discussion

Under tightly controlled experimental conditions, we observed a negative linear relationship between BMI and whole striatal D2BP measured using [^11^C]raclopride in adults across a broad range of adiposity. These findings align with the influential study of Wang and colleagues who also used [^11^C]raclopride to measure D2BP [1]. However, there were no significant associations between BMI and whole striatal D2BP determined by [^18^F]fallypride despite moderate within-subject correlations between the [^11^C]raclopride and [^18^F]fallypride measurements of whole striatal D2BP. Furthermore, the correlation coefficients between BMI and striatal D2BP measured using the two tracers were significantly different from each other.

Given that both [^11^C]raclopride and [^18^F]fallypride PET scans were conducted on the same inpatient subjects in matched physiological states, our findings suggest that the use of different radiotracers may partly explain the inconsistent results over the last two decades concerning the relationship between striatal D2BP and adiposity in humans [29]. The differing kinetics of [^11^C]raclopride and [^18^F]fallypride could potentially explain the discordant associations between adiposity and striatal D2BP and may shed light on striatal dopamine physiology and its connection to human obesity.

For instance, our results might be explained by considering the binding kinetics of [^11^C]raclopride and [^18^F]fallypride for dopamine D2 and D3 receptors. Notably, [^11^C]raclopride binds relatively similarly to both D2 and D3 receptors whereas [^18^F]fallypride has a higher selectivity for D2 receptors [31]. Thus, the observed lack of significant linear correlation between BMI and striatal D2BP measured with [^18^F]fallypride, as opposed to the negative correlation seen with [^11^C]raclopride, could potentially stem from the presence of fewer D3 receptors along with a relatively consistent number of D2 receptors with increasing BMI. However, this explanation faces two challenges. First, D2 receptors are more abundant than D3 receptors within the striatum [32], making it unlikely for a decrease in striatal D3 receptors with BMI to be detected by [^11^C]raclopride. Second, such an effect would likely be most apparent in the ventral striatum, where D3 receptors are relatively more abundant compared to the dorsal striatum [33]. However, the opposite was true, with the negative correlation between BMI and D2BP as measured by [^11^C]raclopride being present in the dorsal striatum, a region with minimal D3 receptors in comparison to D2 receptors [34].

We believe that the most likely interpretation of our data is that adiposity is associated with increased dopamine tone and this relationship may be particularly prominent in the dorsal striatum. Compared to [^18^F]fallypride [21], the [^11^C]raclopride tracer has a lower affinity for the D2 receptor [22] making it more easily displaced by endogenous dopamine [35]. Thus, increased dopamine tone at higher levels of adiposity would be expected to result in a more negative correlation between adiposity and D2BP as measured by [^11^C]raclopride as compared with [^18^F]fallypride. While our calculated estimate of dopamine tone indicated a positive linear relationship with adiposity, it is important to note that our study lacks direct measures of dopamine.

The absence of a significant association between adiposity and striatal D2BP measured with [^18^F]fallypride does not necessarily imply that D2 receptor number is unrelated to adiposity. Despite its high affinity for the D2 receptor, [^18^F]fallypride is somewhat displaceable by manipulations which may increase endogenous dopamine [25, 36, 37]. Therefore, we cannot definitively rule out a positive association between adiposity and striatal D2 receptor number because increased dopamine tone at higher levels of adiposity might mask this relationship by displacing [^18^F]fallypride and result in a more shallow slope not significantly different from zero. Nevertheless, there is evidence to suggest that adiposity may not be related to D2 receptor density as either predicted by Taq1A polymorphism [38] or as quantified using a D2 receptor-specific radiotracer not displaceable by endogenous dopamine [9, 39].

We did not find supporting evidence for Horstman et al.’s hypothesis that the relationship between striatal D2BP and BMI is quadratic [29]. However, our exploratory analyses suggested that percent body fat was quadratically related to D2BP as assessed by either [^18^F]fallypride or [^11^C]raclopride and striatal dopamine tone was positively related with adiposity, but the mechanisms remain uncertain. Tonic dopamine is estimated to occupy between 8–21% of D2R [40] and levels are proportional to the number of active DA terminals and spontaneous, irregular single-spike firing activity of midbrain DA neurons [41, 42]. There is early indication that capacity to synthesize dopamine may be blunted in obesity [43–45]. However, it may be that a limited synthetic capacity impairs the ability to mount a phasic dopamine response to reward-predicting stimuli as opposed to limiting basal dopamine tone in humans [46, 47], particularly in the context of obesity. It is intriguing to speculate that an increase in spontaneous activity of dopamine neurons or number of active terminals may be due proximally to alterations in inhibitory influence from GABAergic inputs [48], or various hormones like ghrelin, leptin, and insulin [49–51].

Of note, our study underscores the largely ignored contribution of recent diet or current metabolic state to D2BP. A recent study pooling 135 individuals studied across protocols using [^11^C]raclopride suggests only a weak association with BMI [19] though it is unclear whether postprandial state or recent diet was ascertained in these cohorts. In exploratory analyses, calculated dopamine tone adjusted for age and sex was negatively correlated to percent of calories from fat in participants’ usual diets (r = −0.391, *p* = 0.007; *n* = 47), which was unrelated to adiposity. Together with evidence that reducing calories specifically from dietary fat increases dopamine tone [25], these exploratory results intriguingly suggest that recent dietary fat intake may have the potential to modulate dopamine tone in humans.

Our study was not designed to elucidate mechanisms, and its cross-sectional design prevents us from determining whether increased adiposity leads to heightened dopamine tone or the reverse. Furthermore, our preplanned analyses were specific to the whole striatum. Given that sub-regional ROI analyses were exploratory and no statistical correction for multiple comparisons was applied, these associations should be confirmed in future studies testing pre-planned hypotheses.

Nevertheless, increased striatal dopamine tone in people with obesity may increase incentive salience and wanting of rewarding stimuli [52]. However, the role of dopamine in the control of food intake and regulation of body weight is complicated. Studies in rodents have identified specific neuron populations in the hypothalamus that control homeostatic food intake that has long been known to influence the brain’s reward system, possibly by augmenting dopamine signaling in the brain’s reward circuitry [53, 54]. Indeed, food restriction increases motivation, incentive salience, and susceptibility to addiction in experimental models [55]. Furthermore, recent studies in mice suggest that brain reward pathways involving dopamine interact with hypothalamic neurons responsible for homeostatic feeding, forming a bidirectional circuit [56]. Exposure of mice to energy-dense and palatable diets affects this bidirectional circuit and leads to excessive food consumption and the development of obesity, while devaluing a non-obesogenic chow diet [57]. Thus, it is possible that alterations in striatal dopamine tone may affect both hedonic and homeostatic control of feeding and may thereby contribute to excess adiposity in people with obesity.

## Methods

Sixty-one adults provided informed consent to participate in a dual PET radiotracer study investigating the relationship between D2R availability and BMI under controlled dietary conditions (ClinicalTrials.gov NCT03648892). Participants were recruited from the community over a wide BMI range and approximately evenly sampled in each of three BMI categories (18.5 kg/m^2^ ≤ BMI < 25 kg/m^2^, 25 kg/m^2^ ≤ BMI < 35 kg/m^2^, BMI ≥ 35 kg/m^2^) to ensure sufficient BMI range to test the quadratic hypothesis. Eligible volunteers were English-speaking, weight stable (less than ± 5% change in the past month), between 18–45 years of age, BMI ≥ 18.5 kg/m^2^. They had no history of bariatric surgery, metabolic disorders, previous traumatic head injury or neurological disorders, severe food allergies (e.g., dairy, gluten) impaired activities of daily living, high blood pressure (>140/90 mm Hg), or current use of medication influencing metabolism or psychiatric medications. They did not have psychiatric conditions or disordered eating (EDE-Q, DSM Cross Cutting Symptom Measure Self Rated Level 1), nicotine dependence, drug use in past 12 months (confirmed via urine toxicology at screening visit), binge drinking over previous 6 months, excessive caffeine consumption, or safety contraindications to MRI. Females were excluded if they were pregnant or lactating. In the women reporting regular menses (not using hormonal contraceptives) (*n* = 31), inpatient admissions started on day 17.4 ± 9.9 of their cycle. Participants self-identified race and ethnicity at the time of admission to the NIH Clinical Center. Handedness was not exclusionary. Participants completed the 10-item Edinburgh Handedness questionnaire to determine laterality quotient [58] and 96.7% of participants (*n* = 59) were determined to be right-handed (laterality quotient > 0).

### Method details

This study was conducted between September 26, 2018 and February 17, 2023. During their inpatient admission, participants completed two PET sessions in pseudorandom order after a confirmed overnight fast. On average, [^18^F]fallypride scans and [^11^C]raclopride scans were completed after 6.5 ± 1.3 and 6.8 ± 1.1 total days of dietary stabilization, respectively. MRI was completed to collect high resolution T-1 weighted structural brain images on which to register individual subject PET data.

The CONSORT diagram provides details of enrollment (Supplementary Fig. 1). No participants withdrew from the inpatient portion after enrollment. Thirty participants received the order of [^11^C]raclopride followed by [^18^F]fallypride while 31 received the order of [^18^F]fallypride followed by [^11^C]raclopride. The initial radiotracer order randomization schedule was provided to the PET Department and was accommodated within the radiotracer production schedule when possible. Of 61 enrolled participants, [^11^C]raclopride scan data are available for *n* = 56 (*n* = 1 participant declined, *n* = 2 scans not performed due to tracer production issue, *n* = 2 scans completed but did not pass quality control on time activity curves) and [^18^F]fallypride scan data are available for *n* = 57 (*n* = 4 scans completed but did not pass quality control on time activity curves). Full PET data ([^11^C]raclopride and [^18^F]fallypride) are available on *n* = 54 participants (Table 1). All participants completed structural MRI.

### Anthropometrics

Height was measured in centimeters using a wall stadiometer (Seca 242, Hanover, MD, USA) and weight was measured in kilograms using a digital scale (Scale-Tronix 5702, Carol Steam, IL, USA). All measurements were obtained after an overnight fast while participants were wearing comfortable clothing.

### Body composition

During the inpatient stay, participants each completed one Dual Energy X-Ray Absorptiometry (DEXA) scan while wearing hospital gown/scrubs to determine body composition (General Electric Lunar iDXA; General Electric; Milwaukee, WI, USA).

### Metabolic diet

Participants were placed on a standard eucaloric diet (50% carbohydrate, 15% protein, 35% fat) with daily energy needs calculated using the Mifflin-St Jeor equation and standard activity factor of 1.5. All meals were prepared in the NIH Clinical Center Nutrition Department Metabolic Kitchen with all foods and beverages weighed on a gram scale (Mettler Toledo Model MS12001L/03).

For the run-in phase, participants were provided with 3–5 days of meals for retrieval from the NIH Clinical Center and consumed them at home prior to admission. Participants were instructed to consume all foods and beverages provided. Any food or beverage not consumed was returned and weighed back. Participants were also instructed to continue their usual caffeine intake in calorie-free forms (e.g., black coffee, diet soda) and abstain from alcohol during this period. For any foods or beverages participants consumed that were not part of the standardized run-in diet, participants were asked to provide a description and amount of what was consumed so that total daily nutrient intake was captured. The eucaloric standardized outpatient diet was provided for an average of 4.5 ± 1.0 days (range 0–5 days). Due to COVID-19 pandemic precautions, one participant was admitted without having completed a diet stabilization, and 3 participants completed some or all of their 3–5 day diet stabilization in the inpatient setting. The remainder (*n* = 57) consumed their stabilization diet as outpatients.

During the inpatient phase, participants continued the same diet and were instructed to consume all foods and beverages provided. All subjects were confined to the metabolic ward throughout their inpatient stay without access to outside food. Meals were consumed under observation. Any uneaten food was weighed back and energy and macronutrients were replaced at the next available meal as needed. Diets were designed using ProNutra software (version 3., Viocare, Inc.). No adverse events, harms or unintended effects resulted from provision of standardized eucaloric diet.

### Questionnaires

The following reflects questionnaire outcomes pertinent to describing participant characteristics. Other exploratory questionnaire outcomes not included will be reported elsewhere. With the exception of the Diet History Questionnaire which was completed directly on the questionnaire website at the initial visit, all questionnaire data were collected at standardized times during the inpatient visit and managed using Research Electronic Data Capture (REDCap) [59, 60] electronic data capture tools hosted at NIDDK.

#### Food frequency questionnaire III (DHQIII; National Cancer Institute)

Participants were instructed to consider intake over the “past year” and report portion sizes consumed. Analyses included variable labeled “Added sugars by total sugar NDSR (grams)”. Outliers were examined across completed questionnaires from all enrolled participants (*n* = 56). We applied a conservative outlier rule to exclude implausible reported intakes (Q3 – (IQR*2.2) = max; Q1 – (IQR*2.2) = min) [30, 61] and three participants were excluded for implausibly high intake. One participant was removed from the analysis for reporting an intake less than 500 kcal/day. A total of 52 eligible dietary histories were eligible for analysis, 47 of which were from participants with both [^11^C]raclopride and [^18^F]fallypride scans available for analysis.

#### Three factor eating questionnaire (TFEQ)

Participants completed a self-assessment questionnaire developed to measure eating behavior traits of dietary restraint, disinhibition and hunger [62].

#### Perceived stress scale (PSS-10)

This questionnaire is designed to assess general psychological stress via appraisal of life events [63]. Participants indicated how often they felt they endorsed 10 prespecified statements over the past month by indicating one of five frequencies (“never”, “almost never”, “sometimes”, “fairly often”, “very often”). Scores range from 0–40 with higher scores indicating higher perceived stress.

#### Beck depression inventory-II

Participants completed a 21-item multiple choice self-report inventory to assess for presence and severity of depressive symptoms [64].

#### Satisfaction with life scale

Participants completed this 5-item scale designed to provide an assessment of life satisfaction that is considered to be relatively stable. Using a 7 point likert scale anchored by “strongly disagree” to “strongly agree”, participants indicated their level of agreement with global cognitive judgments about life satisfaction [65]. Scores range from 5–35 and higher scores indicate greater life satisfaction.

#### Pittsburg sleep quality index

Participants responded to questions about their usual sleep habits during the month leading up to their admission to assess sleep quality [66]. Global sleep quality score ranges from 0–21 and reflects the sum of seven component scores, where higher scores indicate worse sleep quality.

### Magnetic resonance imaging

During their inpatient stay, high resolution anatomical brain MRI was acquired for each subject. Due to the duration of data collection, extended by the COVID-19 pandemic, T1 weighted structural MRIs were collected on 3T Siemens Verio (*n* = 21; TE = 2.98 ms, TR = 2.3 ms, TI = 900 ms, flip angle 9°, slice thickness = 1.2 mm, voxel size 1*1*1.2 mm), and on 3T GE MR-750 Discovery scanner (*n* = 6, TE = 3.04 ms, TR = 7.648 ms, TI = 1060 ms, flip angle 8°, slice thickness = 1.0 mm, voxel size 1*1*1 mm; *n* = 32, TE = 3.46 ms, TR = 8.156 ms, TI = 900 ms, flip angle 7°, slice thickness = 1.0 mm, voxel size 1*1*1 mm) for each subject. Quality of individual subject data were checked by study team [VLD & JG].

The anatomical images were parcellated with FreeSurfer software to generate ROI binary mask volumes in each subject in the putamen, caudate, accumbens, pallidum, and the cerebellum (reference region) (http://surfer.nmr.mgh.harvard.edu). All individual ROI masks were visually checked.

### Positron emission tomography

All PET scanning was performed using a High-Resolution Research Tomograph (HRRT), (Siemens Healthcare, Malvern, PA), a dedicated brain PET scanner with resolution of 2.5–3.0 mm and a 25 cm axial field of view. Transmission scanning was performed with a ^137^Cs rotating point source scan to correct for attenuation. After an overnight fast matched in duration within subject, a bolus of either approximately 5 mCi of [^18^F]fallypride or 20 mCi of [^11^C]raclopride was infused intravenously using a Harvard® pump in semirandom order as discussed above.

The molar activity of [^18^F]fallypride was approximately 7459 mCi/µmol and the radiochemical purity of the radiotracer was > 90%. PET emission data for [^18^F]fallypride were collected starting at radiotracer injection over 3.5 h, in three blocks separated by two 10-min breaks. Thirty-three frames were acquired in list mode at times 0, 0.25, 0.5, 0.75, 1, 1.25, 1.5, 1.75, 2, 2.5, 3, 3.5, 4, 4.5, 5, 6, 7, 8, 9, 10, 12.5, 15, 20, 25, 30, 40, 50, 60, 90, 110, 130, 170, 200 min. The molar activity of [^11^C]raclopride was approximately 4865 mCi/µmol and the radiochemical purity of the radiotracer was > 90%. PET emission data for [^11^C]raclopride were collected starting at radiotracer injection over one block lasting 75 min. Twenty-four frames were acquired in list mode at times 0, 0.5, 1, 1.5, 2.0, 2.5, 3, 4, 5, 6, 8, 10, 15, 20, 25, 30, 35, 40, 45, 50, 55, 60, 65, 70 min. During each scan block, the room was illuminated and quiet, and each subject was instructed to keep their head as still as possible, relax, and try to avoid falling asleep. The image reconstruction process corrected for head motion which was tracked throughout each scan using an optical head tracking sensor (Polaris Vicra, Northern Digital Inc., Shelburne, VT, USA).

Each scan consisted of 207 slices (slice separation = 1.2 mm). The fields of view were 31.2 and 25.2 cm for transverse and axial slices, respectively. The PET images were aligned within each scan block with 6-parameter rigid registration using 7th order polynomial interpolation and each block was aligned to the volume taken at 20 min of the first block. The final alignments were visually checked, with translations varying by < 5 mm and the rotations by < 5°.

For region of interest analyses, individual participants’ anatomical MRI images were co-registered to the aligned PET images by minimizing a mutual information cost function for each individual participant. Time-activity curves for each tracer concentration in the Freesurfer-generated ROIs were extracted and kinetic parameters were fit to a two-compartment model (with the cerebellum used as the reference tissue given negligible D2/3R specific binding [67] to determine regional D2BP [68].

For voxelwise analyses, each individual’s anatomical MRI was nonlinearly transformed into the Talairach space using AFNI 3dQwarp, and the transformation matrix was applied to the PET images which were then smoothed with a 5-mm full-width, half-max Gaussian kernel. Final coregistration was visually checked. Data were exported from Talairach space to MATLAB where time-activity curves for tracer concentration in each voxel were fit to a kinetic model using the cerebellum as a reference tissue to determine D2BP at each voxel and exported back to Talairaich space for group level spatial analyses.

### Statistics

Power calculations were performed with computer simulation to detect a quadratic relationship between D2BP and BMI with 80% of power and 5% of type I error. Based on the review by Horstmann et al. [29], we assumed a quadratic effect of −0.029 m^4^/kg^2^ and a linear effect of 1.913 m^2^/kg. Equal numbers of BMI’s were randomly drawn from three normal distributions (mean ± SD: 21.75 ± 3.15; 30 ± 4.3; 40 ± 4.3 kg/m^2^) to represent the three BMI strata used in the experimental design. The D2BP value for each simulated subject was calculated from its BMI value plus normally-distributed noise (SD = 4). The parameters for the BMI and noise distributions were derived from our previous study [23]. This simulated sample was analyzed using regression analysis and the *p*-value for the quadratic term was calculated. These simulations were repeated 10,000 times and the percentage of *p*-values less than 0.05 determined the power to detect a significant quadratic effect. Our computer simulation suggested that a minimum of 39 subjects (13 per BMI strata) were required to detect a quadratic relationship between BMI and D2BP. This sample size would also be sufficient to provide 89% power to detect a moderate linear association (r = ± 0.45) with a slope of magnitude ≥0.25 m^2^/kg between BMI and D2BP in caudate and putamen using [^18^F]fallypride. Finally, this sample size would also provide >80% power to detect a correlation of r >0.4 between the binding potential of two DA D2 receptor antagonists [^11^C]raclopride and [^18^F]fallypride. Our recruitment exceeded the minimum sample size requirement.

In the ROI analyses, associations between either BMI or percent body fat and D2BP were evaluated with regression analyses. Person correlation coefficients were also reported. Supplementary regression analyses include adiposity (BMI or percent body fat) variables adjusted for sex and age. For associations between D2BP_raclo_ and D2BP_fally_ within ROIs, major axis regressions were also conducted to account for potential measurement error in D2BP calculated from both tracers. An index of regional dopamine tone for was calculated by using the linear relationship between [^11^C]]raclopride and [^18^F]fallypride D2BP in each region to calculate the individual [^11^C]raclopride D2BP adjusted for [^18^F]fallypride D2BP. Because high dopamine tone is expected to result in low levels of adjusted [^11^C]raclopride D2PB, our positive index of regional dopamine done was calculated by subtracting the individual value of the adjusted [^11^C]raclopride D2BP from the maximal group value for that region. Statistical analyses were performed using IBM SPSS Statistics (Version 28.0.1.1, Chicago, IL, USA).

In the voxel-wise analyses, regional clusters where D2BP’s are highly correlated with BMI were identified with regression analysis in AFNI’s 3dttest + + (https://afni.nimh.nih.gov/). Since high D2BP occurs mainly in striatum, small volume corrections were implemented within each hemisphere where D2BP > 1.5. A bi-sided uncorrected voxel-wise threshold of *p* < 0.1 was used (faces touching) to define clusters. Resultant clusters were deemed to survive correction for multiple comparisons using 3dClustSim at alpha of < 0.05 and a threshold of 34 voxels. Voxelwise analyses of correlations between D2BP_raclo_ adjusted by D2BP_fally_ within the striatal mask.

## Supplementary information

Supplement

## Data Availability

Data from consenting individual subjects are available for download at the Open Science Framework (https://osf.io/z23xt/).

## References

[1] Wang GJ, Volkow ND, Logan J, Pappas NR, Wong CT, Zhu W, et al. Brain dopamine and obesity. Lancet. 2001;357:354–7. 11210998 10.1016/s0140-6736(00)03643-6

[2] Johnson PM, Kenny PJ. Dopamine D2 receptors in addiction-like reward dysfunction and compulsive eating in obese rats. Nat Neurosci. 2010;13:635–41. 20348917 10.1038/nn.2519 PMC2947358

[3] Caravaggio F, Raitsin S, Gerretsen P, Nakajima S, Wilson A, Graff-Guerrero A. Ventral striatum binding of a dopamine D 2/3 receptor agonist but not antagonist predicts normal body mass index. Biol Psychiatry. 2014;77:196–202. 10.1016/j.biopsych.2013.02.017 PMC3783412 23540907

[4] Gaiser EC, Gallezot J-D, Worhunsky PD, Jastreboff AM, Pittman B, Kantrovitz L, et al. Elevated dopamine D2/3 receptor availability in obese individuals: a PET imaging study with [11C](+)PHNO. Neuropsychopharmacology. 2016;41:3042–50. 27374277 10.1038/npp.2016.115 PMC5101552

[5] Dunn JP, Kessler RM, Feurer ID, Volkow ND, Patterson BW, Ansari MS, et al. Relationship of dopamine type 2 receptor binding potential with fasting neuroendocrine hormones and insulin sensitivity in human obesity. Diabetes Care. 2012;35:1105–11. 22432117 10.2337/dc11-2250 PMC3329842

[6] Dang LC, Samanez-Larkin GR, Castrellon JJ, Perkins SF, Cowan RL, Zald DH. Associations between dopamine D2 receptor availability and BMI depend on age. Neuroimage. 2016;138:176–83. 27208860 10.1016/j.neuroimage.2016.05.044 PMC4927378

[7] de Weijer BA, van de Giessen E, van Amelsvoort TA, Boot E, Braak B, Janssen IM, et al. Lower striatal dopamine D2/3 receptor availability in obese compared with non-obese subjects. EJNMMI Res. 2011;1:37. 22214469 10.1186/2191-219X-1-37 PMC3265412

[8] Kessler RM, Zald DH, Ansari MS, Li R, Cowan RL. Changes in dopamine release and dopamine D2/3 receptor levels with the development of mild obesity. Synapse. 2014;68:317–20. 24573975 10.1002/syn.21738

[9] Eisenstein SA, Antenor-Dorsey JAV, Gredysa DM, Koller JM, Bihun EC, Ranck SA, et al. A comparison of D2 receptor specific binding in obese and normal-weight individuals using PET with (N-[11C]methyl)benperidol. Synapse. 2013;67:748–56. 23650017 10.1002/syn.21680 PMC3778147

[10] Steele KE, Prokopowicz GP, Schweitzer MA, Magunsuon TH, Lidor AO, Kuwabawa H, et al. Alterations of central dopamine receptors before and after gastric bypass surgery. Obes Surg. 2010;20:369–74. 19902317 10.1007/s11695-009-0015-4

[11] Karlsson HK, Tuominen L, Tuulari JJ, Hirvonen J, Parkkola R, Helin S, et al. Obesity is associated with decreased μ-opioid but unaltered dopamine D2 receptor availability in the brain. J Neurosci. 2015;35:3959–65. 25740524 10.1523/JNEUROSCI.4744-14.2015 PMC6605573

[12] Cho SS, Yoon EJ, Kim SE. Asymmetry of dopamine D2/3 receptor availability in dorsal putamen and body mass index in non-obese healthy males. Exp Neurobiol. 2015;24:90–4. 25792873 10.5607/en.2015.24.1.90 PMC4363338

[13] Janssen LK, Horstmann A. Molecular imaging of central dopamine in obesity: a qualitative review across substrates and radiotracers. Brain Sci. 2022;12:486. 35448017 10.3390/brainsci12040486 PMC9031606

[14] Barry RL, Byun NE, Williams JM, Siuta MA, Tantawy MN, Speed NK, et al. Brief exposure to obesogenic diet disrupts brain dopamine networks. PLoS ONE. 2018;13:e0191299. 29698491 10.1371/journal.pone.0191299 PMC5919534

[15] Friend DM, Devarakonda K, O’Neal TJ, Skirzewski M, Papazoglou I, Kaplan AR, et al. Basal ganglia dysfunction contributes to physical inactivity in obesity. Cell Metab. 2017;25:312–21. 28041956 10.1016/j.cmet.2016.12.001 PMC5299005

[16] Huang X-F, Yu Y, Zavitsanou K, Han M, Storlien L. Differential expression of dopamine D2 and D4 receptor and tyrosine hydroxylase mRNA in mice prone, or resistant, to chronic high-fat diet-induced obesity. Brain Res Mol Brain Res. 2005;135:150–61. 15857678 10.1016/j.molbrainres.2004.12.013

[17] Sharma S, Fulton S. Diet-induced obesity promotes depressive-like behaviour that is associated with neural adaptations in brain reward circuitry. Int J Obes. 2013;37:382–9. 10.1038/ijo.2012.48 22508336

[18] Ong ZY, Wanasuria AF, Lin MZP, Hiscock J, Muhlhausler BS. Chronic intake of a cafeteria diet and subsequent abstinence. Sex-specific effects on gene expression in the mesolimbic reward system. Appetite. 2013;65:189–99. 23402719 10.1016/j.appet.2013.01.014

[19] Malén T, Karjalainen T, Isojärvi J, Vehtari A, Bürkner P-C, Putkinen V, et al. Atlas of type 2 dopamine receptors in the human brain: age and sex dependent variability in a large PET cohort. Neuroimage. 2022;255:119149. 35367652 10.1016/j.neuroimage.2022.119149

[20] Pak K, Nummenmaa L. Brain dopamine receptor system is not altered in obesity: bayesian and frequentist meta-analyses. Hum Brain Mapp. 2023;44:6552–60. 37950852 10.1002/hbm.26534 PMC10681634

[21] Mukherjee J, Yang Z-Y, Das MK, Brown T. Fluorinated benzamide neuroleptics—III. development of (S)-N-[(1-allyl-2-pyrrolidinyl)methyl]-5-(3-[18F]fluoropropyl)-2,3-dimethoxybenzamide as an improved dopamine D-2 receptor tracer. Nucl Med Biol. 1995;22:283–96. 7627142 10.1016/0969-8051(94)00117-3

[22] Köhler C, Hall H, Ögren S-O, Gawell L. Specific in vitro and in vivo binding of 3H-raclopride a potent substituted benzamide drug with high affinity for dopamine D-2 receptors in the rat brain. Biochem Pharmacol. 1985;34:2251–9. 4015674 10.1016/0006-2952(85)90778-6

[23] Guo J, Simmons WK, Herscovitch P, Martin A, Hall KD. Striatal dopamine D2-like receptor correlation patterns with human obesity and opportunistic eating behavior. Mol Psychiatry. 2014;19:1078–84. 25199919 10.1038/mp.2014.102 PMC4189966

[24] Guo J, Simmons WK, Herscovitch P, Martin A, Hall KD. Correction to: Striatal dopamine D2-like receptor correlation patterns with human obesity and opportunistic eating behavior. Mol Psychiatry. 2022;27:4369. 36104439 10.1038/s41380-022-01767-5

[25] Darcey VL, Guo J, Courville AB, Gallagher I, Avery JA, Simmons WK, et al. Dietary fat restriction affects brain reward regions in a randomized crossover trial. JCI Insight. 2023;8:e169759. 37345661 10.1172/jci.insight.169759 PMC10371234

[26] Volkow ND, Wang GJ, Fowler JS, Logan J, Gatley SJ, MacGregor RR, et al. Measuring age-related changes in dopamine D2 receptors with 11C-raclopride and 18F-N-methylspiroperidol. Psychiatry Res. 1996;67:11–6. 8797238 10.1016/0925-4927(96)02809-0

[27] Wong DF, Wagner HN, Dannals RF, Links JM, Frost JJ, Ravert HT, et al. Effects of age on dopamine and serotonin receptors measured by positron tomography in the living human brain. Science. 1984;226:1393–6. 6334363 10.1126/science.6334363

[28] Williams OOF, Coppolino M, George SR, Perreault ML. Sex differences in dopamine receptors and relevance to neuropsychiatric disorders. Brain Sci. 2021;11:1199. 34573220 10.3390/brainsci11091199 PMC8469878

[29] Horstmann A, Fenske WK, Hankir MK. Argument for a non-linear relationship between severity of human obesity and dopaminergic tone. Obes Rev. 2015;16:821–30. 26098597 10.1111/obr.12303

[30] Hoaglin DC, Iglewicz B. Fine-tuning some resistant rules for outlier labeling. J Am Stat Assoc. 1987;82:1147–9.

[31] Hatano K, Ishiwata K, Elsinga HP. PET tracers for imaging of the dopaminergic system. Curr Med Chem. 2006;13:2139–53. 16918344 10.2174/092986706777935258

[32] Lahti RA, Roberts RC, Tamminga CA. D2-family receptor distribution in human postmortem tissue: an autoradiographic study. Neuroreport. 1995;6:2505–12. 8741751 10.1097/00001756-199512150-00015

[33] Tziortzi AC, Searle GE, Tzimopoulou S, Salinas C, Beaver JD, Jenkinson M, et al. Imaging dopamine receptors in humans with [11C]-(+)-PHNO: dissection of D3 signal and anatomy. Neuroimage. 2011;54:264–77. 20600980 10.1016/j.neuroimage.2010.06.044

[34] Seeman P, Wilson A, Gmeiner P, Kapur S. Dopamine D2 and D3 receptors in human putamen, caudate nucleus, and globus pallidus. Synapse. 2006;60:205–11. 16739118 10.1002/syn.20298

[35] Laruelle M. Imaging synaptic neurotransmission with in vivo binding competition techniques: a critical review. J Cereb Blood Flow Metab. 2000;20:423–51. 10724107 10.1097/00004647-200003000-00001

[36] Riccardi P, Li R, Ansari MS, Zald D, Park S, Dawant B, et al. Amphetamine-induced displacement of [18F] fallypride in striatum and extrastriatal regions in humans. Neuropsychopharmacology. 2006;31:1016–26. 16237395 10.1038/sj.npp.1300916

[37] Cropley VL, Innis RB, Nathan PJ, Brown AK, Sangare JL, Lerner A, et al. Small effect of dopamine release and no effect of dopamine depletion on [18F]fallypride binding in healthy humans. Synapse. 2008;62:399–408. 18361438 10.1002/syn.20506

[38] Benton D, Young HA. A meta-analysis of the relationship between brain dopamine receptors and obesity: a matter of changes in behavior rather than food addiction? Int J Obes. 2016;40:S12–21. 10.1038/ijo.2016.9 PMC4819757 27001642

[39] Moerlein SM, Perlmutter JS, Markham J, Welch MJ. In vivo kinetics of [18F](N-methyl)benperidol: a novel PET tracer for assessment of dopaminergic D2-like receptor binding. J Cereb Blood Flow Metab. 1997;17:833–45. 9290581 10.1097/00004647-199708000-00002

[40] Caravaggio F, Iwata Y, Kim J, Shah P, Gerretsen P, Remington G, et al. What proportion of striatal D2 receptors are occupied by endogenous dopamine at baseline? A meta-analysis with implications for understanding antipsychotic occupancy. Neuropharmacology. 2020;163:107591. 30940535 10.1016/j.neuropharm.2019.03.034

[41] Grace AA. Dysregulation of the dopamine system in the pathophysiology of schizophrenia and depression. Nat Rev Neurosci. 2016;17:524–32. 27256556 10.1038/nrn.2016.57 PMC5166560

[42] Floresco SB, West AR, Ash B, Moore H, Grace AA. Afferent modulation of dopamine neuron firing differentially regulates tonic and phasic dopamine transmission. Nat Neurosci. 2003;6:968–73. 12897785 10.1038/nn1103

[43] Wilcox CE, Braskie MN, Kluth JT, Jagust WJ. Overeating behavior and striatal dopamine with 6–Fluoro-L–Tyrosine PET. J Obes. 2010;2010:1–6. 10.1155/2010/909348 PMC2925447 20798859

[44] Wallace DL, Aarts E, Dang LC, Greer SM, Jagust WJ, D’Esposito M. Dorsal striatal dopamine, food preference and health perception in humans. PLoS ONE. 2014;9:1–7. 10.1371/journal.pone.0096319 PMC4012945 24806534

[45] Lee Y, Kroemer NB, Oehme L, Beuthien B, Goschke T, Smolka MN. Lower dopamine tone in the striatum is associated with higher body mass index. Eur Neuropsychopharmacol. 2018;28:719–31. 29705023 10.1016/j.euroneuro.2018.03.009

[46] Ito H, Kodaka F, Takahashi H, Takano H, Arakawa R, Shimada H, et al. Relation between presynaptic and postsynaptic dopaminergic functions measured by positron emission tomography: implication of dopaminergic tone. J Neurosci. 2011;31:7886–90. 21613502 10.1523/JNEUROSCI.6024-10.2011 PMC6633123

[47] Berry AS, Shah VD, Furman DJ, White RL, Baker SL, O’Neil JP, et al. Dopamine synthesis capacity is associated with D2/3 receptor binding but not dopamine release. Neuropsychopharmacology. 2018;43:1201–11. 28816243 10.1038/npp.2017.180 PMC5916345

[48] Tepper JM, Lee CR. GABAergic control of substantia nigra dopaminergic neurons. Prog Brain Res. 2007;160:189–208. 17499115 10.1016/S0079-6123(06)60011-3

[49] Andrews ZB, Erion D, Beiler R, Liu ZW, Abizaid A, Zigman J, et al. Ghrelin promotes and protects nigrostriatal dopamine function via a UCP2-dependent mitochondrial mechanism. J Neurosci. 2009;29:14057–65. 19906954 10.1523/JNEUROSCI.3890-09.2009 PMC2845822

[50] Kullmann S, Blum D, Assad Jaghutriz B, Gassenmaier C, Bender B, Häring H-U, et al. Central insulin modulates dopamine signaling in the human striatum. J Clin Endocrinol Metab. 2021;106:2949–61. 34131733 10.1210/clinem/dgab410

[51] Hagan MM, Havel PJ, Seeley RJ, Woods SC, Ekhator NN, Baker DG, et al. Cerebrospinal fluid and plasma leptin measurements: covariability with dopamine and cortisol in fasting humans. J Clin Endocrinol Metab. 1999;84:3579–85. 10522999 10.1210/jcem.84.10.6034

[52] Wise RA. Dual roles of dopamine in food and drug seeking: the drive-reward paradox. Biol Psychiatry. 2013;73:819–26. 23044182 10.1016/j.biopsych.2012.09.001 PMC3548035

[53] Reichenbach A, Clarke RE, Stark R, Lockie SH, Mequinion M, Dempsey H, et al. Metabolic sensing in AgRP neurons integrates homeostatic state with dopamine signalling in the striatum. eLife. 2022;11:e72668. 35018884 10.7554/eLife.72668 PMC8803314

[54] van der Plasse G, van Zessen R, Luijendijk MC, Erkan H, Stuber GD, Ramakers GM, et al. Modulation of cue-induced firing of ventral tegmental area dopamine neurons by leptin and ghrelin. Int J Obes. 2015;39:1742–9. 10.1038/ijo.2015.131 PMC4722241 26183405

[55] Carr KD. Modulatory effects of food restriction on brain and behavioral effects of abused drugs. Curr Pharm Des. 2020;26:2363–71. 32013842 10.2174/1381612826666200204141057 PMC8905183

[56] Alhadeff AL, Goldstein N, Park O, Klima ML, Vargas A, Nicholas Betley J. Natural and drug rewards engage distinct pathways that converge on coordinated hypothalamic and reward circuits HHS public access. Neuron. 2019;103:891–908. 31277924 10.1016/j.neuron.2019.05.050 PMC6728176

[57] Mazzone CM, Liang-Guallpa J, Li C, Wolcott NS, Boone MH, Southern M, et al. High-fat food biases hypothalamic and mesolimbic expression of consummatory drives. Nat Neurosci. 2020;23:1253–66. 32747789 10.1038/s41593-020-0684-9 PMC7529959

[58] Oldfield RC. The assessment and analysis of handedness: the Edinburgh inventory. Neuropsychologia. 1971;9:97–113. 5146491 10.1016/0028-3932(71)90067-4

[59] Harris PA, Taylor R, Thielke R, Payne J, Gonzalez N, Conde JG. Research electronic data capture (REDCap)—A metadata-driven methodology and workflow process for providing translational research informatics support. J Biomed Inform. 2009;42:377–81. 18929686 10.1016/j.jbi.2008.08.010 PMC2700030

[60] Harris PA, Taylor R, Minor BL, Elliott V, Fernandez M, O’Neal L, et al. The REDCap consortium: building an international community of software platform partners. J Biomed Inform. 2019;95:103208. 31078660 10.1016/j.jbi.2019.103208 PMC7254481

[61] Burcham S, Liu Y, Merianos AL, Mendy A. Outliers in nutrient intake data for U.S. adults: national health and nutrition examination survey 2017–2018. Epidemiol Methods. 2023;12:20230018. 38013683 10.1515/em-2023-0018 PMC10637781

[62] Stunkard AJ, Messick S. The three-factor eating questionnaire to measure dietary restraint, disinhibition and hunger. J Psychosom Res. 1985;29:71–83. 3981480 10.1016/0022-3999(85)90010-8

[63] Cohen S Perceived stress in a probability sample of the United States. 1988.

[64] Beck AT, Steer RA, Brown GK. Beck depression inventory (BDI-II): manual and questionnaire. San Antonio, TX: The Psychological Corporation; 1996.

[65] Diener E, Emmons R, Larsen R, Griffin S. The satisfaction with life scale. J Pers Assess. 1985;49:71–5. 16367493 10.1207/s15327752jpa4901_13

[66] Buysse DJ, Reynolds CF 3rd, Monk TH, Berman SR, Kupfer DJ. The Pittsburgh sleep quality index: a new instrument for psychiatric practice and research. Psychiatry Res. 1989;28:193–213. 2748771 10.1016/0165-1781(89)90047-4

[67] Vandehey NT, Moirano JM, Converse AK, Holden JE, Mukherjee J, Murali D, et al. High-affinity dopamine D2/D3 PET radioligands 18F-fallypride and 11C-FLB457: a comparison of kinetics in extrastriatal regions using a multiple-injection protocol. J Cereb Blood Flow Metab. 2010;30:994–1007. 20040928 10.1038/jcbfm.2009.270 PMC2897717

[68] Lammertsma AA, Hume SP. Simplified reference tissue model for PET receptor studies. Neuroimage. 1996;4:153–8. 9345505 10.1006/nimg.1996.0066

