# Supplementary material for: Striatal dopamine tone is positively associated with adiposity in humans as determined by PET using dual dopamine type-2 receptor antagonist tracers
Source: Mol Psychiatry. 2025 Apr 6;30(8):3708–17. doi: 10.1038/s41380-025-02960-y (PMC12240848; doi:10.1038/s41380-025-02960-y)
Supplement: Supplementary file 1 — Supplement [file 41380_2025_2960_MOESM1_ESM.pdf]

SUPPLEMENTARY DATA

Striatal dopamine tone is positively associated with adiposity in humans as determined by PET using dual dopamine type-2 receptor antagonist tracers

Valerie L. Darcey, Juen Guo, Meible Chi, Stephanie T. Chung, Amber B. Courville, Isabelle Gallagher, Peter Herscovitch, Rebecca Howard, Melissa La Noire, Lauren Milley, Alex Schick, Michael Stagliano, Sara Turner, Nicholas Urbanski, Shanna Yang, Eunha Yim, Nan Zhai, Megan S. Zhou, Kevin D. Hall

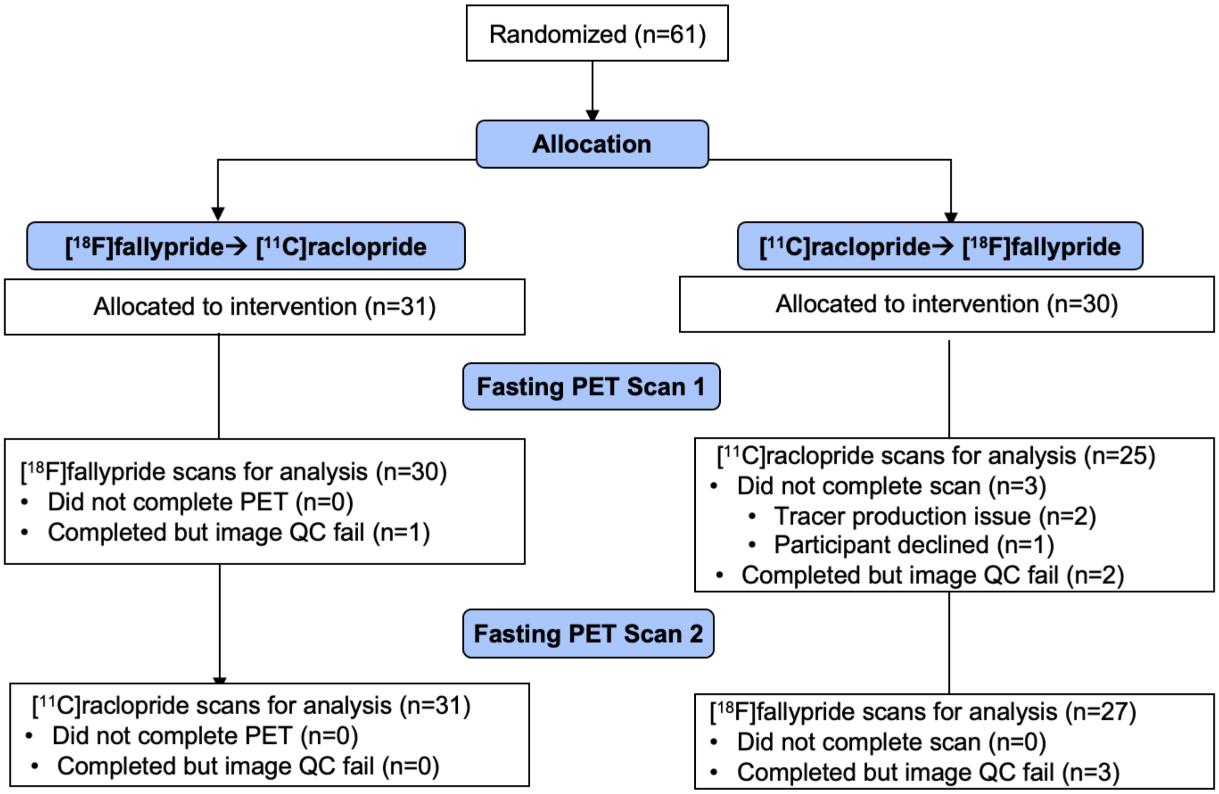

Supplementary Figure 1. CONSORT diagram.

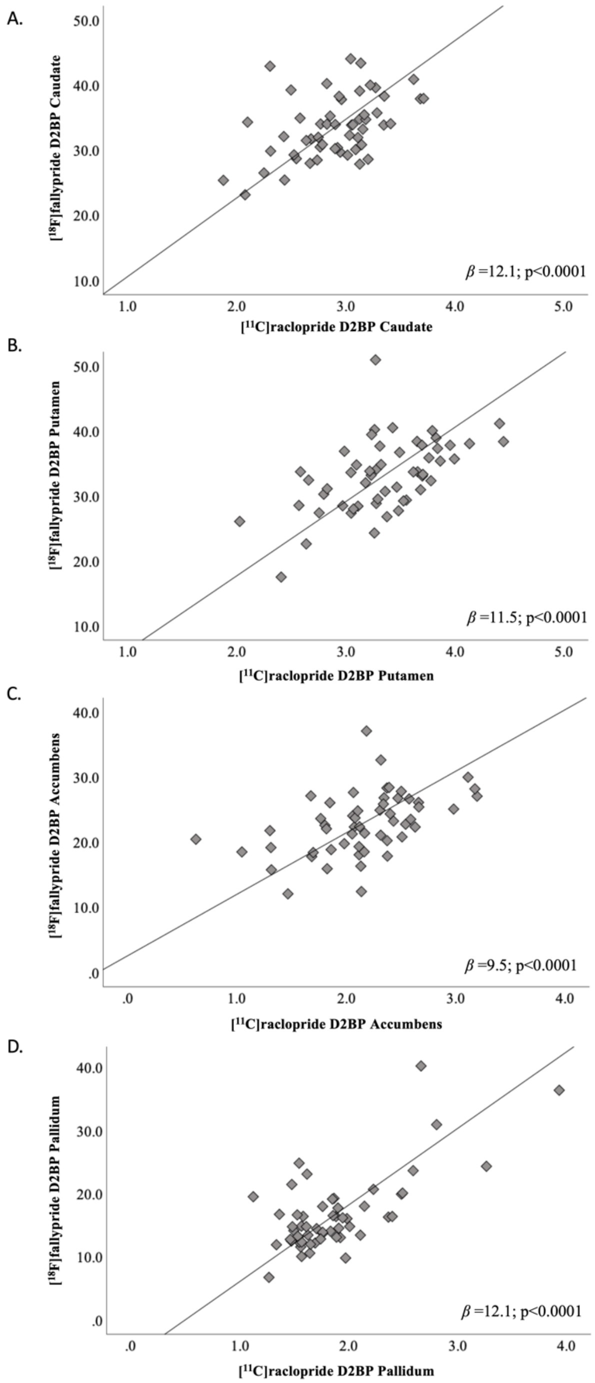

Supplementary Figure 2. Relationship between $D2BP_{raclo}$ and $D2BP_{fally}$ within bilateral striatal regions of interest. Striatal regions of interest analyses in n=54 participants with valid scans using both tracers in (A) caudate, (B) putamen, (C) accumbens, and (D) pallidum. Trendline and slope parameters reflect standard major axis regression. Not corrected for multiple comparisons.

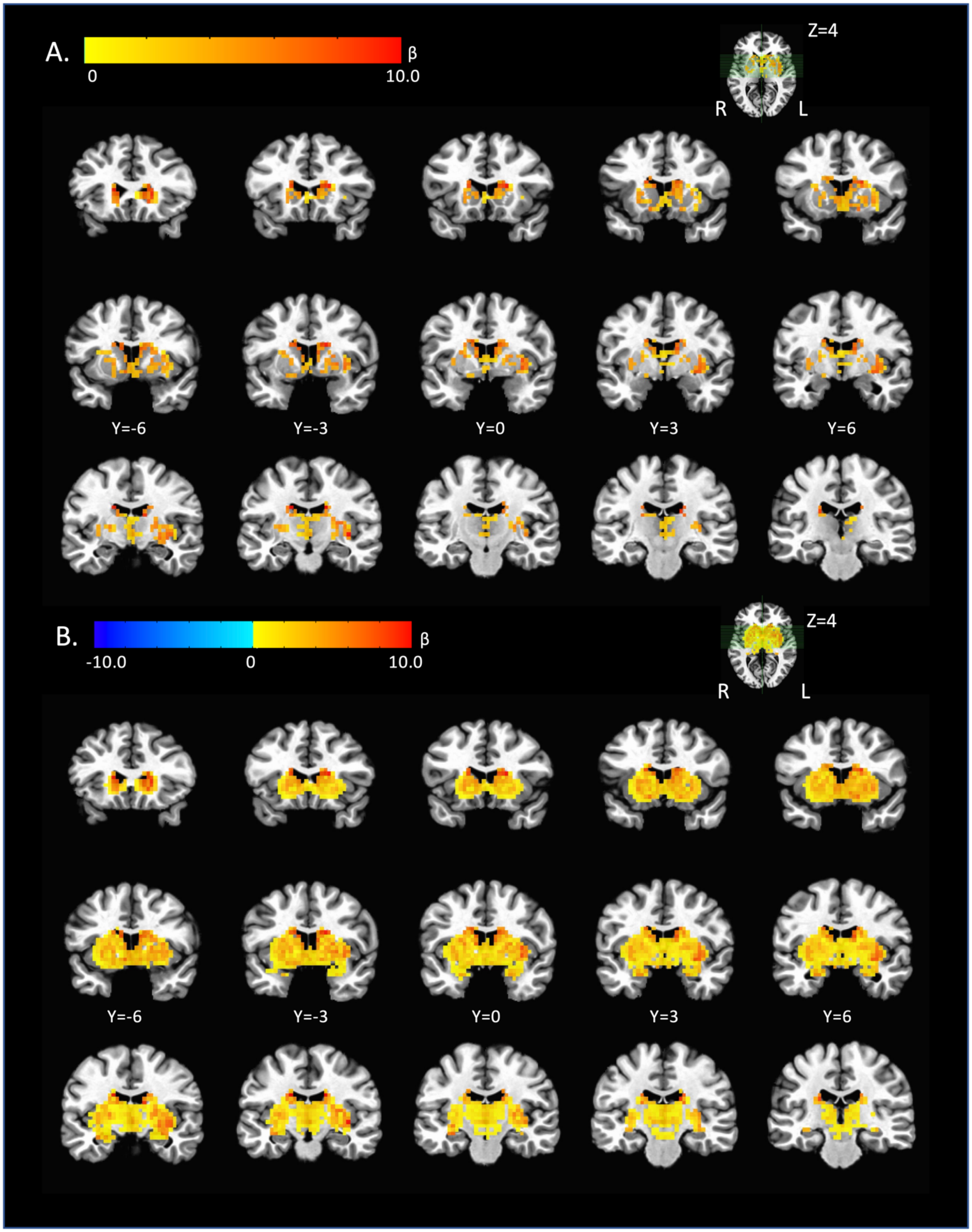

Supplementary Figure 3. Voxelwise analysis of correlation between $D2BP_{raclo}$ and $D2BP_{fally}$ across n=54 participants. (A) Thresholded. (B) Unthresholded maps.

Supplementary Table 1. Participant usual diet, usual sleep quality, trait characteristics including eating behavior and mood.

	Enrolled Participants (N)	Enrolled participants	[^{11}C]raclopride & [^{18}F]fallypride (N)	[^{11}C]raclopride & [^{18}F]fallypride
Usual energy intake (kcal/day)	52	1497 ± 662	47	1500 ± 660
Protein (% kcal)	52	15.7 ± 4.2	47	15.9 ± 4.1
Fat, total (%kcal)	52	33.0 ± 8.2	47	33.6 ± 8.3
Saturated fat (%kcal)	52	10.5 ± 3.0	47	10.6 ± 3.1
Fatty acid ratio (unsat:sat)	52	1.9 ± 0.4	47	2.0 ± 0.4
Carbohydrate, total (% kcal)	52	51.6 ± 11.7	47	50.8 ± 11.7
Added sugars (grams)	52	47.0 ± 40.3	47	42.3 ± 32.3
**Three Factor Eating Questionnaire**				
Cognitive Restraint	61	8.3 ± 4.7	54	8.2 ± 4.5
Disinhibition	61	4.8 ± 2.7	54	4.9 ± 2.7
Hunger	61	3.2 ± 2.6	54	3.3 ± 2.7
**Perceived Stress Scale**				
Mean	60	9.4 ± 5.6	53	9.6 ± 5.6
Mode(s)	60	6, 7	53	7
Range	60	0 – 22	53	0 – 22
**Beck Depression Inventory**				
Mean	61	1.9 ± 2.6	54	2.0 ± 2.7
Mode	61	0	54	0
Range	61	0 – 10	54	0 – 10
**Satisfaction with Life Scale**				

Mean	60	26.4 ± 4.9	53	26.1 ± 4.9
Mode	60	27	53	27
Range	60	11 – 35	53	11 – 35
**Pittsburgh Sleep Quality Index**				
Mean	47	4.4 ± 2.1	41	4.6 ± 2.1
Mode	47	3	41	3
Range	47	1 – 10	41	1 – 10

Supplementary Table 2. Locations of striatal clusters with significant correlations. PET resolution 3.5mm^3. Imaging analyses conducted in Analysis of Functional Neuroimaging (AFNI) within striatal region binding potential mask (clusters defined by voxels with faces touching).

	**Location of peak**			**Voxels**	**Size (mm^3)**	**t-stat** $^{(f)}$	**alpha**
	x	y	z				
$D2BP_{fally}$ x $D2BP_{raclo}$ $^{(a)}$							
Left caudate, posterior	15.8	11.5	24.0	371	15907	8.86	<0.01
Left caudate, anterior	15.8	-20.0	10.0	94	4030	5.29	<0.01
Right putamen, insula	-36.8	11.5	-0.5	125	5359	3.16	<0.01
$D2BP_{fally}$ x BMI $^{(b)}$							
Right caudate	-12.2	-9.5	3.0	36	1544	1.8	<0.05
Right thalamus	-1.8	8.0	6.5	28	1201	-3.4	>0.10
Left caudate	12.2	-6.0	6.5	27	1158	3.7	>0.10
$D2BP_{raclo}$ x BMI $^{(c)}$							
Left putamen	29.8	8.0	3.0	172	7375	-4.10	<0.01
Right putamen	-29.8	8.0	-0.5	162	6946	-4.16	<0.01
$D2BP_{raclo\ (adjusted\ by\ D2BP\text{-}fally)}$ x BMI $^{(d)}$							
Right putamen	-29.8	8.0	-0.5	285	12220	4.33	<0.01
Left putamen	29.8	8.0	3.0	167	7161	4.16	<0.01
Voxelwise difference in $D2BP_{raclo}$ vs BMI and $D2BP_{fally}$ vs BMI correlation coefficients $^{(e)}$							
Left caudate/putamen	8.8	1.0	6.5	58	2487	-0.63	<0.01
Right globus pallidus	-15.8	8.0	3.0	29	1243	-0.64	>0.10
Right caudate/putamen	-12.2	-6.0	3.0	26	1115	-0.46	>0.10

a. 1 sample t-test, n=54, k_e=20, $p_{uncorrected}$ = 0.01
b. 1 sample t-test, n=57, k_e=20, $p_{uncorrected}$ = 0.1
c. 1 sample t-test, n=56, k_e=20, $p_{uncorrected}$ = 0.1
d. 1 sample t-test, n=54, k_e=20, $p_{uncorrected}$ = 0.1
e. Paired samples t-test, n=54, k_e=20, $p_{uncorrected}$ = 0.01
f. For (e), value reflects peak difference between correlation coefficients

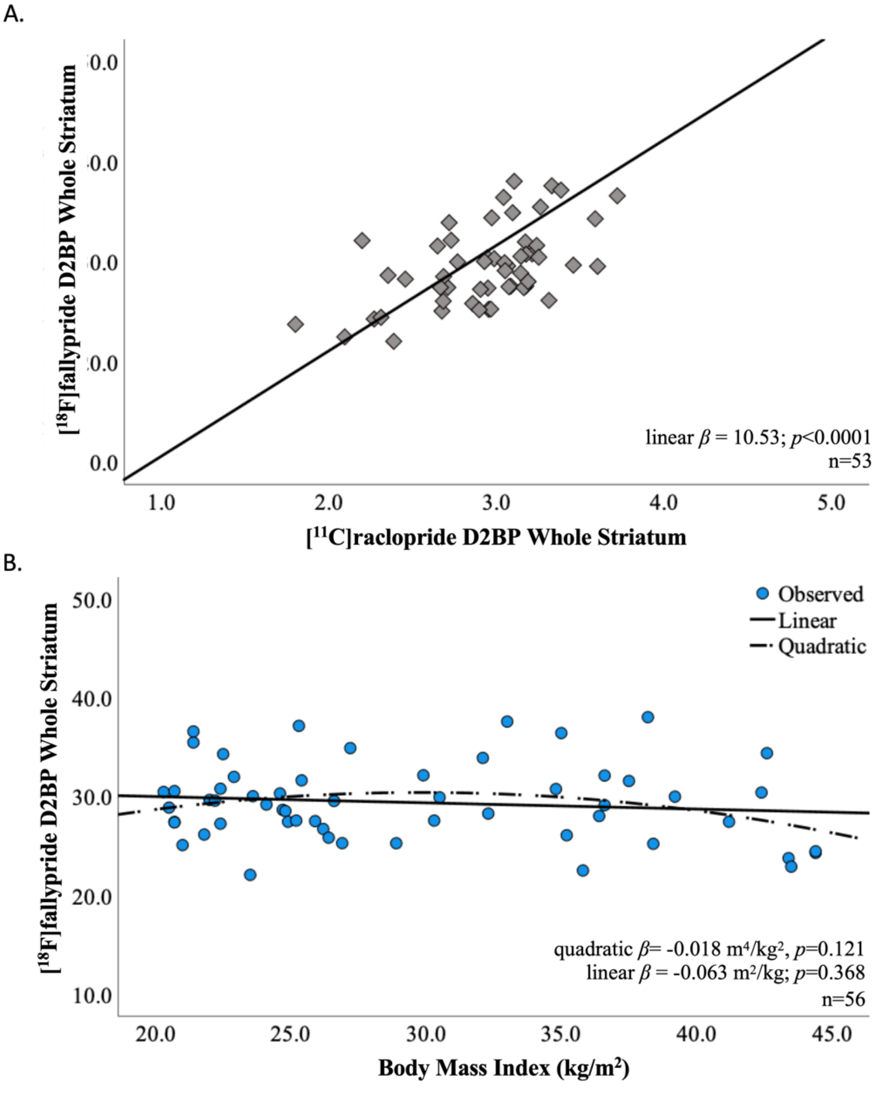

Supplementary Figure 4. Re-analysis of primary aims exclusive of one outlier [^{18}F] fallypride datapoint. Primary reported conclusions are robust to inclusion or exclusion of outlier. (A) Relationship between D2BP measurements using [^{11}C]raclopride and [^{18}F]fallypride excluding one outlier datapoint (n=53) (B) and relationship between D2BP measured by [^{18}F]fallypride and BMI (n=56).

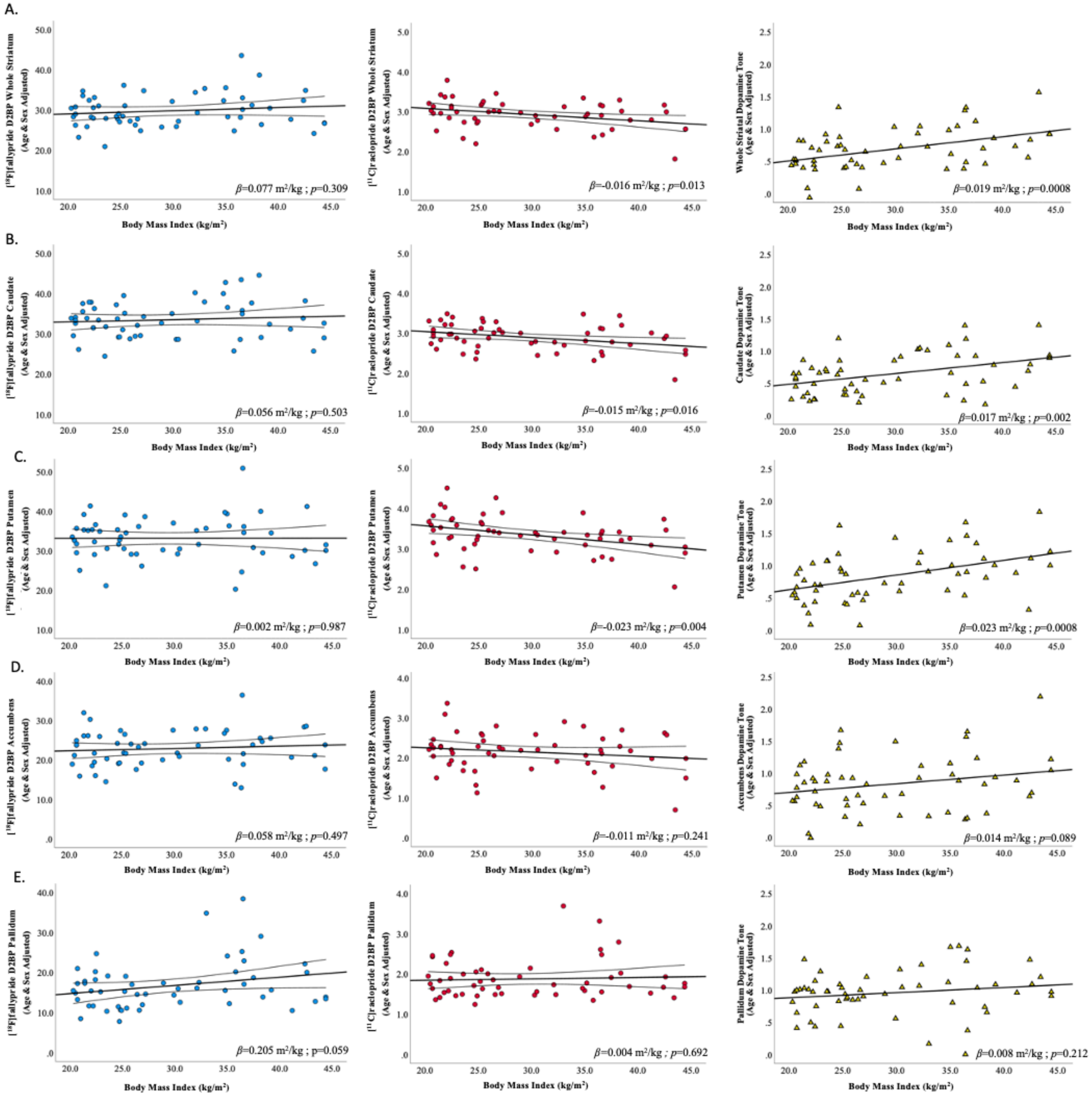

Supplementary Figure 5. Relationship between BMI and D2BP and an index of dopamine tone adjusted for age and sex for 54 participants with both tracers as measured by [^{18}F]fallypride (blue circles, left column) and [^{11}C]raclopride (red circles, center column) across (A) whole striatum, (B) caudate, (C) putamen, (D) accumbens, and (E) pallidum. Regression line and 95%CI indicated. Relationship between BMI and an index of dopamine tone (yellow triangles, right column) adjusted for age and sex across ROIs. Uncorrected for multiple comparisons.

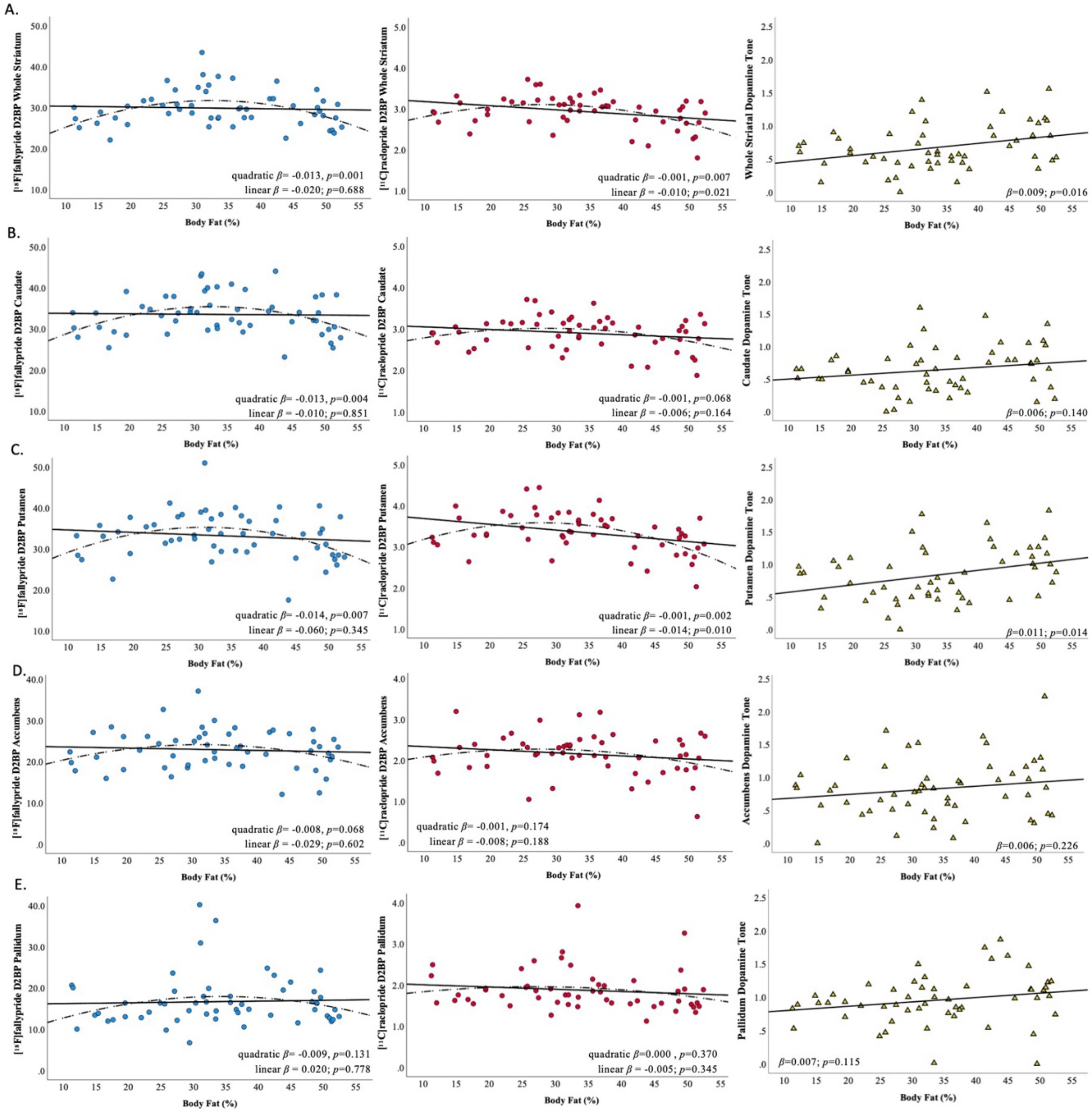

Supplementary Figure 6. Relationship between percent body fat and D2BP and an index of dopamine tone for 54 participants as measured by [^{18}F]fallypride (blue circles, left column) and [^{11}C]raclopride (red circles, center column) across (A) whole striatum, (B) caudate, (C) putamen, (D) accumbens, and (E) pallidum. Relationship between percent body fat and index of dopamine tone (yellow triangles, right column) across ROIs. Uncorrected for multiple comparisons.

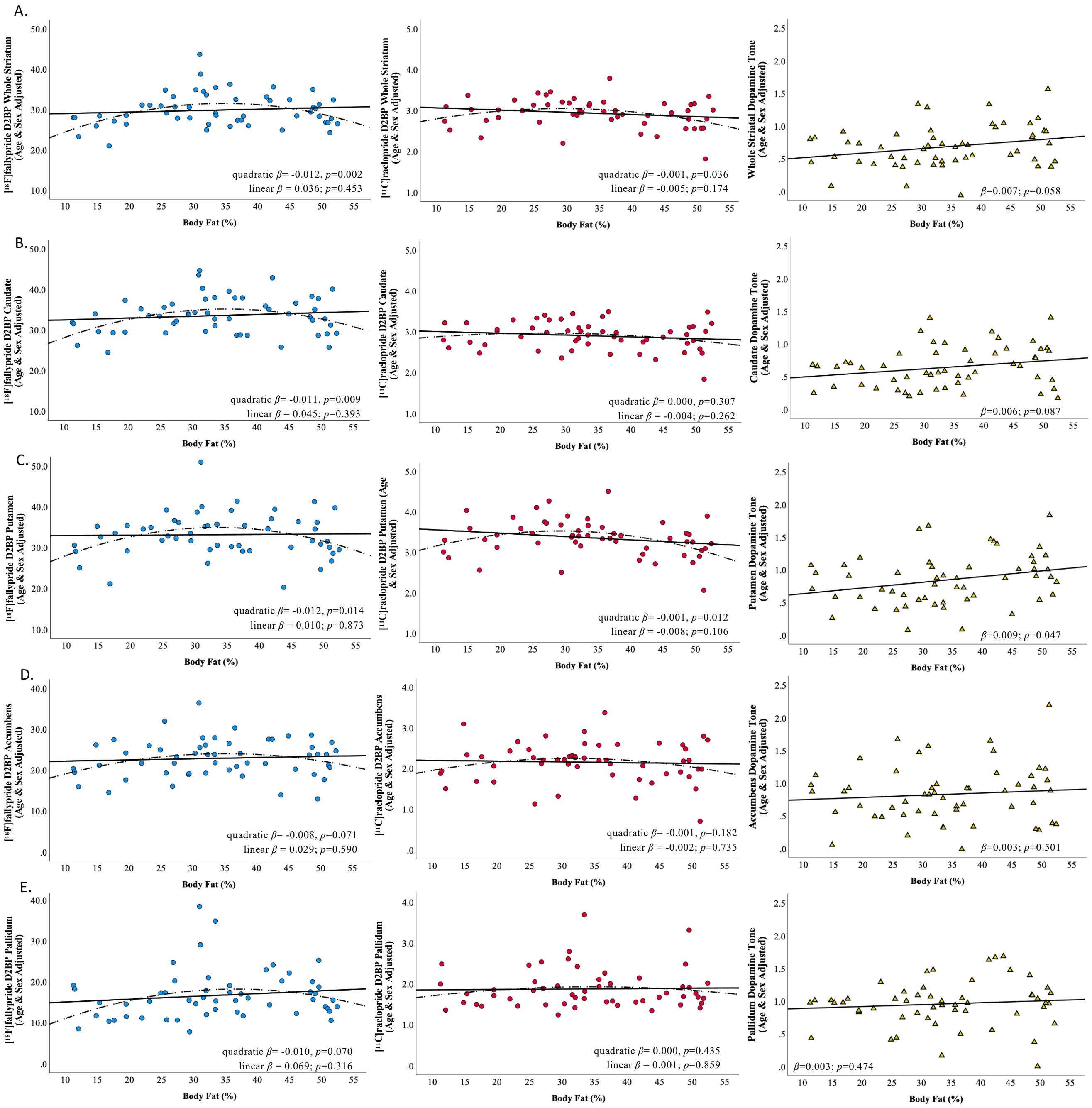

Supplementary Figure 7. Relationship between percent body fat and D2BP and an index of dopamine tone adjusted for age and sex for 54 participants as measured by [^{18}F]fallypride (blue circles, left column) and [^{11}C]raclopride (red circles, center column) across (A) whole striatum, (B) caudate, (C) putamen, (D) accumbens, and (E) pallidum. Relationship between percent body fat and dopamine tone (yellow triangles, right column) adjusted for age and sex across ROIs. Uncorrected for multiple comparisons.

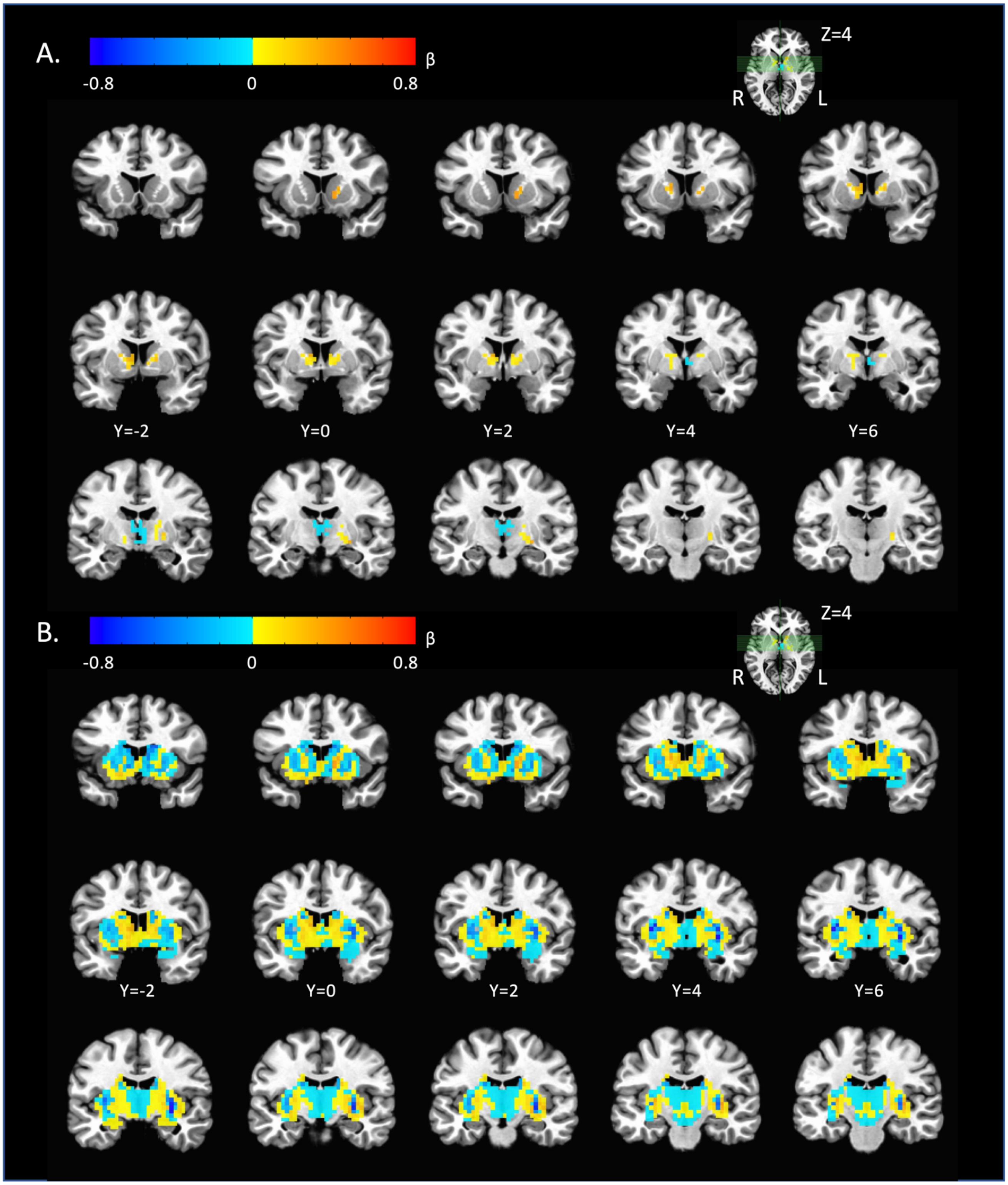

Supplementary Figure 8. Voxelwise analysis of relationship between D2BP$_{fally}$ and BMI across n=57 participants. (A) Thresholded (See Supplementary Table 2 for peak coordinates) (B) Unthresholded maps.

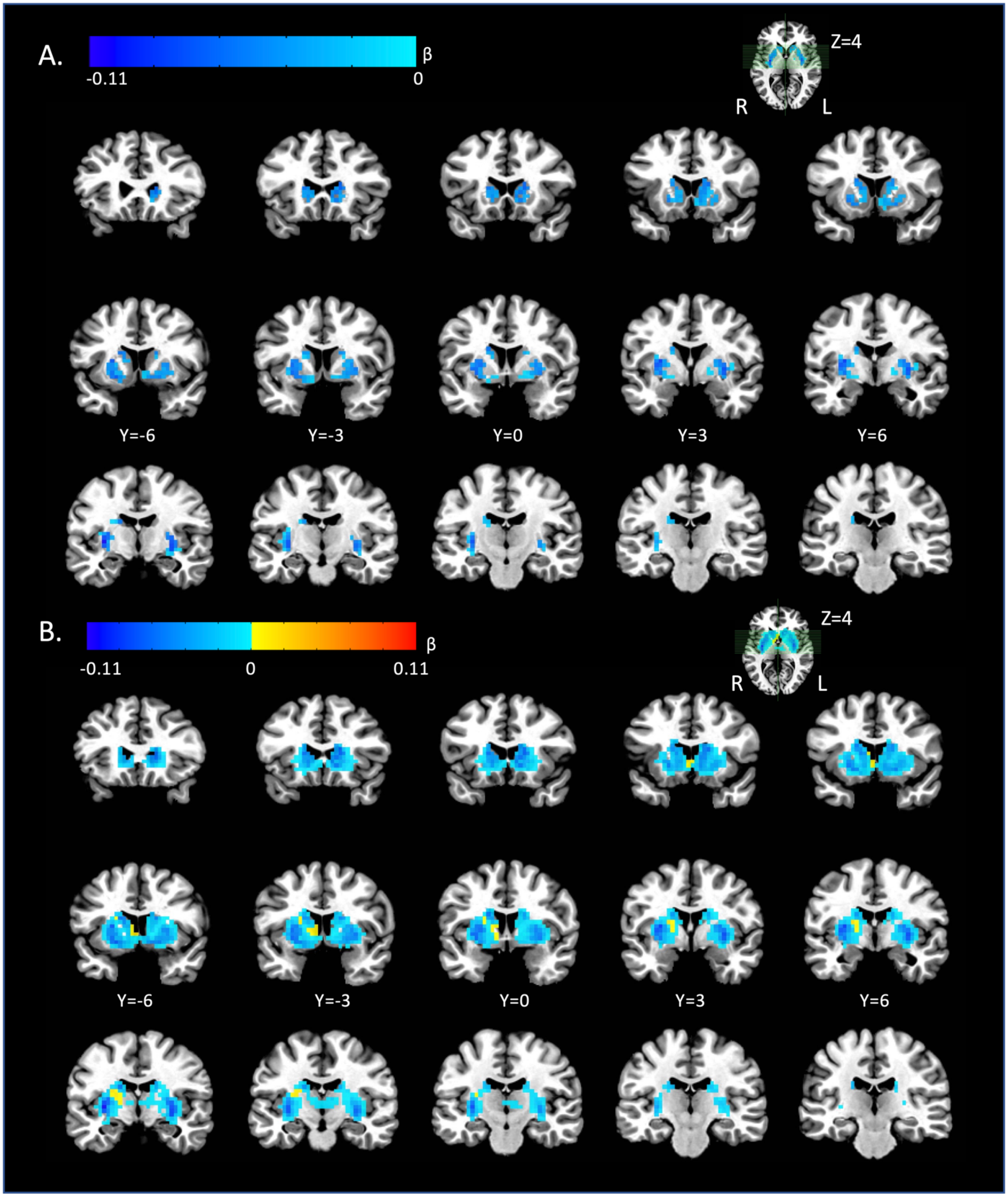

Supplementary Figure 9. Voxelwise analysis of relationship between D2BP$_{raclo}$ and BMI across n=56 participants. (A) Thresholded. (See Supplementary Table 2 for peak coordinates.) (B) Unthresholded maps.

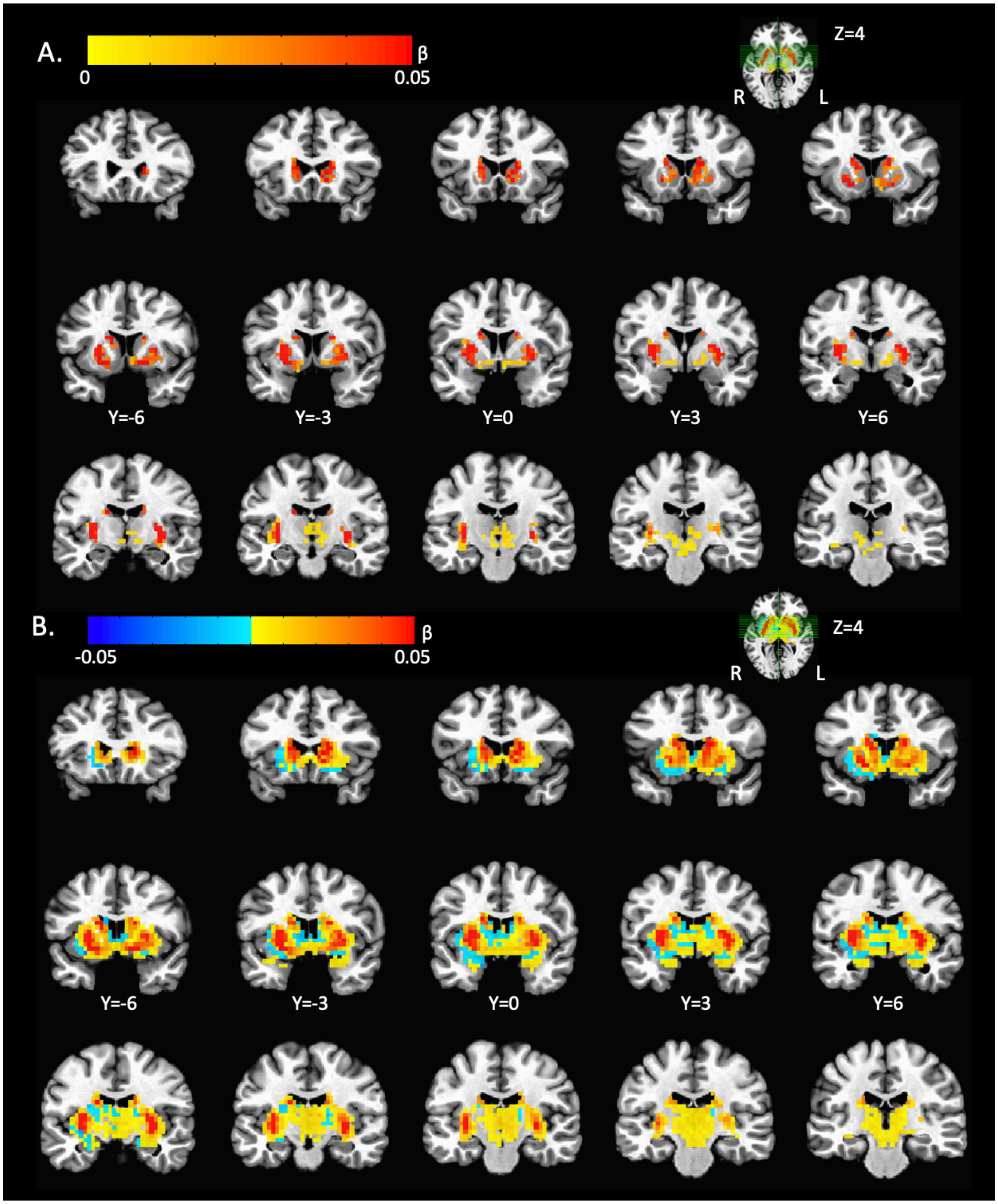

Supplementary Figure 10. Voxelwise analysis of relationship between an index of dopamine tone and BMI across n=54 participants. (A) Thresholded. (See Supplementary Table 2 for peak coordinates.) (B) Unthresholded maps.

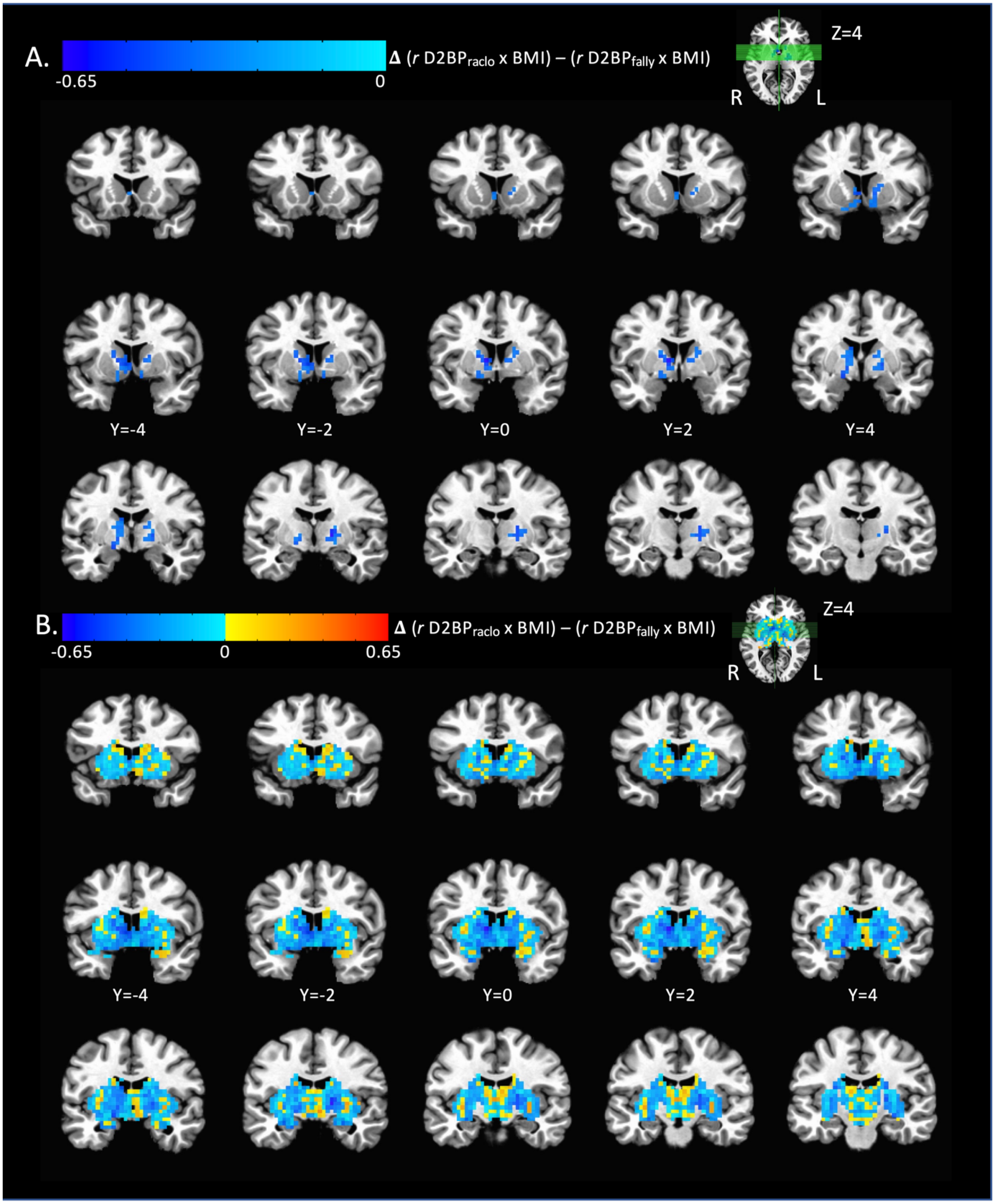

Supplementary Figure 11. Voxelwise analysis of difference between normalized correlation coefficient for D2BP$_{raclo}$ x BMI and normalized correlation coefficient for D2BP$_{fally}$ x BMI across n=54 participants. (A) Thresholded. (See Supplementary Table 2 for peak coordinates.) (B) Unthresholded maps.
